# Diazoxide for Severe or Recurrent Neonatal Hypoglycemia: A Randomized Clinical Trial

**DOI:** 10.1001/jamanetworkopen.2024.15764

**Published:** 2024-06-13

**Authors:** Don Laing, Eamon P. G. Walsh, Jane M. Alsweiler, Sara M. Hanning, Michael P. Meyer, Julena Ardern, Wayne S. Cutfield, Jenny Rogers, Gregory D. Gamble, J. Geoffrey Chase, Jane E. Harding, Christopher J. D. McKinlay

**Affiliations:** 1 Liggins Institute, University of Auckland, Auckland, New Zealand; 2 Department of Paediatrics: Child and Youth Health, University of Auckland, Auckland, New Zealand; 3 Te Whatu Ora Te Toka Tumai Auckland, Auckland, New Zealand; 4 School of Pharmacy, University of Auckland, Auckland, New Zealand; 5 Kidz First Neonatal Care, Te Whatu Ora Counties Manukau, Auckland, New Zealand; 6 Department of Mechanical Engineering, University of Canterbury, Christchurch, New Zealand

## Abstract

**Importance:**

Neonatal hypoglycemia is an important preventable cause of neurodevelopmental impairment, but there is a paucity of evidence to guide treatment.

**Objective:**

To evaluate whether early, low-dose oral diazoxide for severe or recurrent neonatal hypoglycemia reduces time to resolution of hypoglycemia.

**Design, Setting, and Participants:**

This 2-arm, placebo-controlled randomized clinical trial was conducted from May 2020 to February 2023 in tertiary neonatal units at 2 New Zealand hospitals. Participants were neonates born at 35 or more weeks’ gestation and less than 1 week of age with severe hypoglycemia (blood glucose concentration <22 mg/dL or <36 mg/dL despite 2 doses of dextrose gel) or recurrent hypoglycemia (≥3 episodes of a blood glucose concentration <47 mg/dL within 48 hours).

**Interventions:**

Newborns were randomized 1:1 to receive diazoxide suspension (loading dose, 5 mg/kg; maintenance, 1.5 mg/kg every 12 hours) or placebo, titrated per protocol.

**Main Outcome and Measures:**

The primary outcome was time to resolution of hypoglycemia, defined as enteral bolus feeding without intravenous fluids and normoglycemia (blood glucose concentration of 47-98 mg/dL) for at least 24 hours, compared between groups using adjusted Cox proportional hazards regression. Hazard ratios adjusted for stratification variables and gestation length are reported. Prespecified secondary outcomes, including number of blood glucose tests and episodes of hypoglycemia, duration of hypoglycemia, and time to enteral bolus feeding and weaning from intravenous fluids, were compared by generalized linear models. Newborns were followed up for at least 2 weeks.

**Results:**

Of 154 newborns screened, 75 were randomized and 74 with evaluable data were included in the analysis (mean [SD] gestational age for the full cohort, 37.6 [1.6] weeks), 36 in the diazoxide group and 38 in the placebo group. Baseline characteristics were similar: in the diazoxide group, mean (SD) gestational age was 37.9 (1.6) weeks and 26 (72%) were male; in the placebo group, mean (SD) gestational age was 37.4 (1.5) weeks and 27 (71%) were male. There was no significant difference in time to resolution of hypoglycemia (adjusted hazard ratio [AHR], 1.39; 95% CI, 0.84-2.23), possibly due to increased episodes of elevated blood glucose concentration and longer time to normoglycemia in the diazoxide group. Resolution of hypoglycemia, when redefined post hoc as enteral bolus feeding without intravenous fluids for at least 24 hours with no further hypoglycemia, was reached by more newborns in the diazoxide group (AHR, 2.60; 95% CI, 1.53-4.46). Newborns in the diazoxide group had fewer blood glucose tests (adjusted count ratio [ACR], 0.63; 95% CI, 0.56-0.71) and episodes of hypoglycemia (ACR, 0.32; 95% CI, 0.17-0.63), reduced duration of hypoglycemia (adjusted ratio of geometric means [ARGM], 0.18; 95% CI, 0.06-0.53), and reduced time to enteral bolus feeding (ARGM, 0.74; 95% CI, 0.58-0.95) and weaning from intravenous fluids (ARGM, 0.72; 95% CI, 0.60-0.87). Only 2 newborns (6%) treated with diazoxide had hypoglycemia after the loading dose compared with 20 (53%) with placebo.

**Conclusions and Relevance:**

In this randomized clinical trial, early treatment of severe or recurrent neonatal hypoglycemia with low-dose oral diazoxide did not reduce time to resolution of hypoglycemia but reduced time to enteral bolus feeding and weaning from intravenous fluids, duration of hypoglycemia, and frequency of blood glucose testing compared with placebo.

**Trial Registration:**

ANZCTR.org.au Identifier: ACTRN12620000129987

## Key Points

**Question:**

Does early, low-dose oral diazoxide for severe or recurrent neonatal hypoglycemia reduce time to resolution of hypoglycemia?

**Findings:**

In this randomized clinical trial of 74 newborns, treatment of severe or recurrent neonatal hypoglycemia with diazoxide did not reduce time to resolution of hypoglycemia, defined as enteral bolus feeding without intravenous fluids and normoglycemia for at least 24 hours.

**Meaning:**

Further investigation of low-dose oral diazoxide is warranted in newborns who do not respond to initial treatment of hypoglycemia with dextrose gel and breastfeeding.

## Teaser

This randomized clinical trial investigates whether administration of early, low-dose oral diazoxide in neonates with severe or recurrent hypoglycemia reduces time to normoglycemia.

## Introduction

Up to 30% of newborns have at least 1 risk factor for hypoglycemia, including preterm birth, maternal diabetes, and fetal undergrowth or overgrowth, and about half of these newborns experience hypoglycemia.^1^ The developing brain is uniquely sensitive to neuroglycopenia, which can cause selective neuronal necrosis and impaired maturation, leading to cognitive deficits and learning difficulties in later childhood,^2^ especially in those exposed to severe hypoglycemia (blood glucose concentration <36 mg/dL; to convert to mmol/L, multiply by 0.0555) or recurrent episodes of hypoglycemia.^3,4^ Thus, neonatal hypoglycemia remains one of the most important and potentially preventable causes of impaired development in newborns.

About 20% of newborns with hypoglycemia require admission to neonatal care when first-line measures, such as buccal dextrose gel, fail to normalize the blood glucose concentration.^3^ Not only are these newborns at increased risk of neurodevelopmental sequelae but they are also exposed to a high burden of medical therapy, including prolonged hospitalization, frequent heel lancing, high rates of formula use, and separation from their mothers. Despite the short- and long-term importance, there is a paucity of high-quality data about how to best manage care for newborns with severe or recurrent hypoglycemia, and there is increasing concern that exogenous glucose could exacerbate the effects of neuroglycopenia.^5^ Therefore, better treatments are needed for these newborns that reduce the burden of care, improve long-term outcomes, and ensure equity for all ethnic groups.^6,7^ Ideally, treatments should target the pathophysiology, especially dysregulated insulin secretion with impairment of hepatic glucose output,^2^ and directly promote metabolic transition.^7^

We undertook the Neonatal Glucose Care Optimisation (NeoGluCO) Study to investigate use of low-dose oral diazoxide in the early management of severe or recurrent hypoglycemia in late preterm through full-term newborns.^8^ In pancreatic β cells, insulin exocytosis is controlled by adenosine triphosphate (ATP)–sensitive potassium channels (K_ATP_), which close as glucose-generated ATP increases, triggering cell membrane depolarization. Diazoxide binds to the K_ATP_ sulfonylurea receptor, causing hyperpolarization and attenuation of glucose-stimulated insulin secretion.^9^ We hypothesized that diazoxide therapy, by modulating β-cell responsiveness to glucose, would improve glycemic stability, allowing earlier weaning from intravenous fluids and establishment of enteral feeding.^8^

## Methods

### Study Design

The NeoGluCO Study (ACTRN12620000129987) was a double-blinded, 2-arm, parallel randomized clinical trial of diazoxide vs placebo (trial protocol in Supplement 1)^8^ conducted in Middlemore Hospital and Auckland City Hospital, New Zealand, from May 2020 to February 2023. Ethical approval was provided by the Central Health and Disability Ethics Committee of New Zealand. Parents or guardians provided prospective written informed consent. Reporting of this trial follows the Consolidated Standards of Reporting Trials (CONSORT) reporting guideline.^10^

### Participants

Eligible newborns were born at 35 or more weeks’ gestation and admitted to the neonatal care unit in the first week with severe hypoglycemia, defined as a blood glucose concentration less than 22 mg/dL, a concentration less than 36 mg/dL despite 2 doses of buccal dextrose gel and feeding in a single episode, or recurrent (≥3) consecutive episodes of blood glucose concentration less than 47 mg/dL in 48 hours.^8^ Exclusion criteria included major congenital malformations, suspected genetic or chromosomal disorders, confirmed sepsis, hypoxic-ischemic encephalopathy, or family history of congenital hyperinsulinism.

### Interventions

Newborns were randomly allocated online (1:1) to diazoxide or placebo. The allocation sequence was computer generated with random permuted blocks of 4 and 6, stratified by center and small-for-gestational-age birth weight (<10th customized centile).^11^ Newborns in the diazoxide intervention group received a loading dose of 5 mg/kg orally or by gastric tube followed by a maintenance dose of 1.5 mg/kg every 12 hours.^12^ Diazoxide was prepared by hospital pharmacists by adding five 100-mg diazoxide capsules to 50 mL of sugar-free pediatric suspension (10 mg/mL).^13^ The placebo intervention was an equal volume of the suspension solution. Blinding was maintained by using opaque bottles labeled with a 4-digit random number and by adding a small amount of cornstarch to the placebo (4 g per 50 mL), making it visibly identical to the diazoxide suspension.^13^ Tetrad testing among hospital staff showed that the interventions had sensory equivalence and could not be distinguished.^8^

The interventions were titrated using a bedside algorithm,^8^ commencing before the third maintenance dose, to a target blood glucose concentration of 47 to 98 mg/dL, representing the 10th and 90th centiles for healthy breastfed newborns in the first week.^14^ Once the primary outcome was reached, 1 further intervention dose was given unless the intervention had already been discontinued according to the algorithm. Intravenous fluids and feeding were provided according to local guidelines, but staff were encouraged to wean from fluids and introduce feeding as soon as possible once blood glucose concentration stabilized.

### Assessments

Ethnicity was determined by self-report and included to provide descriptive statistics; categories included Indian, Māori, Middle Eastern, European New Zealander, and Pacific. Blood glucose concentrations were measured by a blood gas analyzer (ABL700; Radiometer) every 6 hours or less (before feeding if bolus feeding) until the primary outcome was reached. Newborns also had continuous interstitial glucose monitoring (Medtronic Guardian Connect, Enlite-3 sensor),^8^ and text alerts were sent to research personnel via remote cloud monitoring; if predefined low or high trend alarm criteria were met, bedside nurses were requested to measure an additional blood glucose concentration.^8^ Once the primary outcome was achieved, interstitial glucose concentration and blood glucose concentration were monitored for 24 hours or longer. Decisions about titration of the intervention and clinical management were based exclusively on blood glucose concentration.

Blood samples were collected before randomization for measurement of blood gas and plasma insulin, β-hydroxybutyrate, free fatty acid, and creatinine concentrations by the hospital laboratory. Additional plasma samples were collected before the third maintenance dose for later assay of diazoxide,^13^ insulin, and creatinine. All newborns had a routine neonatal metabolic screening by Guthrie test at 48 or more hours. At Middlemore Hospital, cardiac ultrasonography was performed 72 or more hours after commencement of the study intervention (echocardiography was only available at Auckland City Hospital for symptomatic newborns). Newborns were followed up clinically for a minimum of 2 weeks or until discharge to home, whichever was longer.

### Outcomes

The primary outcome was time to resolution of hypoglycemia, defined as the point at which all of the following occurred concurrently for at least 24 hours: enteral bolus feeding, normoglycemia (blood glucose concentration of 47-98 mg/dL), and no intravenous fluids.^8^ Secondary efficacy outcomes included time to achieve normoglycemia, enteral bolus feeding and full sucking feeding for at least 24 hours, feeding at discharge, use of intravenous fluids and type, duration of intravenous fluids, hypoglycemia (frequency, duration, and timing), number of blood glucose tests, and duration of admission.^8^ Secondary safety outcomes included commencement of low-flow oxygen or positive pressure respiratory support; congestive heart failure; patent ductus arteriosus, pulmonary hypertension, and cardiac impairment on ultrasonography; frequency, duration, and timing of elevated blood glucose concentration (99-124 mg/dL) or hyperglycemia (≥125 mg/dL); discontinuation of the study intervention due to elevated blood glucose concentration or hyperglycemia or another adverse event; hypoglycemia after discontinuation of the study intervention; seizures; death; inborn errors of metabolism; and plasma insulin, creatinine, and diazoxide concentrations.^8^

### Statistical Analysis

Analysis was performed with SAS, version 9.4 (SAS Institute Inc) using a statistical analysis plan prepared before completion of recruitment (Statistical Analysis Plan in Supplement 1). All analyses were prespecified unless otherwise stated, and outcomes were calculated before unblinding. Treatment groups were compared for the primary outcome using Cox proportional hazards regression analysis adjusted for stratification variables (site and customized birth weight centile) and gestation length, with planned censoring at 4 weeks. The primary treatment effect is presented as an adjusted hazard ratio (AHR) with a 95% CI. Secondary outcomes were compared between treatment groups using generalized linear models appropriate for the dependent variable, adjusted for stratification variables and gestation length. Secondary treatment effects are presented as the adjusted mean difference (AMD), adjusted ratio of the geometric mean (ARGM), adjusted odds ratio (AOR), or adjusted count ratio (ACR), as appropriate, with 95% CIs.

We estimated that 74 newborns (37 per group) would give 80% power to detect a relative hazard of 2.0, equivalent to a 2-day difference to reach the primary outcome, assuming that at least 90% of newborns in each group reached that point (2-tailed α = .05) (PASS, version 16 [NCSS]).^15^ An adaptive sample size approach was adopted in which the number of randomized participants was increased by the number of participants who withdrew.

## Results

**Figure 1.**
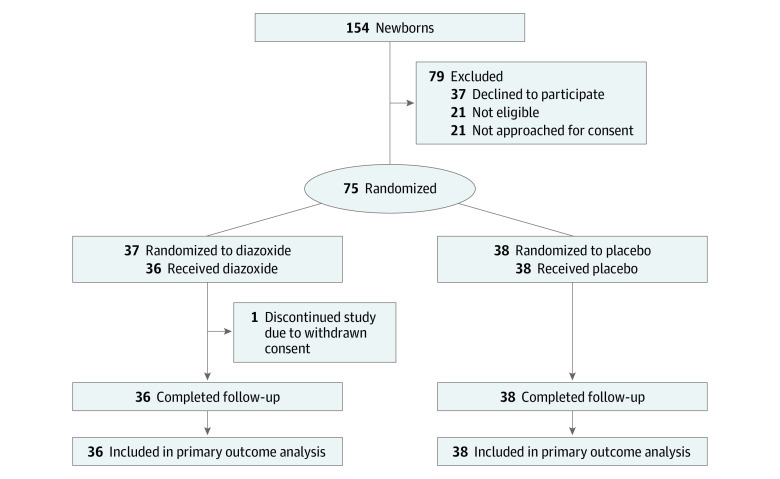
Participant Flow in the Neonatal Glucose Care Optimisation Study

During recruitment, 154 newborns were screened, of whom 133 (86%) met eligibility criteria; parents of 112 newborns were approached for consent, which was obtained for 75 newborns (67%), all of whom were randomized (diazoxide: n = 37; placebo: n = 38) (Figure 1). Consent for 1 newborn in the diazoxide group was withdrawn after randomization but before commencement of the intervention; no outcome data were available for this newborn. Thus, 74 newborns were included in the analysis (mean [SD] gestational age for the full cohort, 37.6 [1.6] weeks). Of these newborns, 9 (12%) were Indian; 7 (9%), Māori; 1 (1%), Middle Eastern; 10 (14%), European New Zealander; and 47 (64%), Pacific. The intervention groups were well balanced for maternal and pregnancy characteristics and newborn demographics. In the diazoxide group, mean (SD) gestational age was 37.9 (1.6) weeks; 10 (28%) were female, and 26 (72%) were male. In the placebo group, mean (SD) gestational age was 37.4 (1.5) weeks; 11 (29%) were female, and 27 (71%) were male. Groups were also well balanced for baseline metabolites, including blood glucose, lactate, insulin, β-hydroxybutyrate, free fatty acid, and creatinine concentrations (Table 1). Overall, 45 newborns (60%) were randomized at less than 24 hours, 19 (25%) from 24 to 48 hours, and 11 (15%) at 48 hours or more.

**Table 1.** Baseline Characteristics of Newborns and Their Mothers in the Neonatal Glucose Care Optimisation Study

Characteristic	Diazoxide group		Placebo group	
	Value^a^	Total No.	Value^a^	Total No.
**Maternal**				
Age, mean (SD), y	30.9 (5.9)	36	30.6 (6.0)	38
Parity				
Previous births after 20 wk gestation, median (IQR), No.	1 (0-3)	36	1 (0-2)	38
Nulliparous	14 (39)		13 (34)	
Height, mean (SD), mo	1.65 (0.07)	36	1.65 (0.06)	38
BMI at first pregnancy visit, mean (SD)	34.8 (8.5)	35	36.0 (8.2)	38
Obesity^b^	24 (69)		28 (76)	
Gestational weight gain, mean (SD), kg	11.2 (5.9)	31	11.4 (6.9)	32
HbA_1c_ at <20 wk, median (IQR), % of total hemoglobin	5.6 (5.3-7.2)	31	5.7 (5.3-7.6)	31
Diabetes				
Any	20 (56)	36	20 (53)	38
Pregestational	12 (34)		15 (39)	
Gestational	8 (22)		5 (13)	
Third-trimester HbA_1c_, median (IQR), % of total hemoglobin^c^	6.5 (6.0-7.2)	19	6.9 (6.6-7.9)	19
Hypertensive disorders of pregnancy	7 (19)	36	14 (37)	38
Previous infant with transitional hypoglycemia	3 (8)	36	4 (11)	38
Planned feeding method				
Breastfeeding	29 (81)	36	26 (68)	38
Breastfeeding and formula	6 (16)		11 (29)	
Formula	1 (3)		1 (3)	
**Neonatal**				
Sex				
Female	10 (28)	36	11 (29)	38
Male	26 (72)		27 (71)	
Twin^d^	2 (6)	36	2 (5)	38
Fetal growth restriction (obstetric diagnosis)	6 (17)	36	4 (11)	38
Cesarean delivery				
Any	24 (67)	36	33 (87)	38
Emergency	17 (47)		24 (63)	
Gestational age, mean (SD), wk	37.9 (1.6)	36	37.4 (1.5)	38
Preterm^e^	10 (28)	36	14 (37)	38
Birth weight				
Mean (SD), g	3419 (803)	36	3444 (885)	38
Customized centile, median (IQR)	70 (5-99)		66 (6-98)	
Size for gestational age, customized centile				
Small, <10	12 (33)	36	12 (32)	38
Large, >90	14 (39)		14 (37)	
Apgar score <7 at 5 min	0	36	5 (11)	38
Prioritized ethnicity				
Indian	3 (8)	36	6 (16)	38
Māori	5 (14)		2 (5)	
Middle Eastern	1 (3)		0	
European New Zealander	5 (14)		5 (13)	
Pacific	22 (61)		25 (66)	
Eligibility criteria^f^				
Recurrent hypoglycemia	24 (65)	36	17 (45)	38
Severe hypoglycemia	9 (24)		12 (32)	
Profound hypoglycemia	12 (33)		19 (50)	
Admission plasma metabolite concentrations				
Age at blood sample attainment, median (IQR), h	17.3 (7.9-25.7)	34	10.5 (4.1-7.8)	35
Blood glucose level, mean (SD), mg/dL	52 (20)	34	49 (20)	35
Lactate level, mean (SD), mg/dL	30.6 (14.4)	33	35.1 (14.4)	34
Insulin level, median (IQR), μIU/mL	1.6 (0.6-2.6)	29	1.9 (0.9-3.2)	33
Insulin-to-glucose ratio, median (IQR), U/mol^g^	4.5 (2.0-7.9)	20	5.2 (2.7-10.8)	22
β-Hydroxybutyrate detected	8 (25)	32	6 (18)	33
β-Hydroxybutyrate level, mean (SD), mmol/L	0.2 (0.1)	8	0.2 (0.1)	6
Free fatty acids, median (IQR), mg/dL	27 (18-53)	28	27 (18-53)	31
Creatinine level, mean (SD), mg/dL	0.84 (0.20)	25	0.84 (0.18)	31
Intravenous dextrose delivery at metabolite testing, mean (SD), mg/kg/min	6.0 (3.1)	19	6.2 (2.9)	21
Age at randomization, median (IQR), h^h^	24.6 (10.9-31.9)	36	18.4 (19.5-26.1)	38
Intravenous dextrose at randomization	25 (69)	36	23 (61)	38

Abbreviations: BMI, body mass index (calculated as weight in kilograms divided by height in meters squared); HbA_1c_, glycated hemoglobin.

SI conversion factors: To convert blood glucose concentration to mmol/L, multiply by 0.0555; creatinine to μmol/L, multiply by 88.4; glycated hemoglobin to proportion of total hemoglobin, multiply by 0.01; insulin to pmol/L, multiply by 6.945; lactate to mmol/L, multiply by 0.111.

^a^ Data are presented as number (percentage) of mothers or neonates unless otherwise indicated.

^b^ Defined as BMI greater than 30.

^c^ Test closest to birth was included in the analysis.

^d^ For newborns from twin pregnancies, no sibling pairs were included.

^e^ Less than 37 weeks’ gestation.

^f^ Not mutually exclusive; all blood glucose concentrations were measured by blood gas analyzer. Recurrent hypoglycemia, 3 or more episodes of blood glucose less than 47 mg/dL in 48 hours; severe, 22 to 36 mg/dL despite 2 doses of dextrose gel and feeding; and profound, 1 or more episodes less than 22 mg/dL.

^g^ Only included if blood glucose concentration was obtained within 15 minutes of insulin sample.

^h^ Randomization was stratified by site and small-for-gestational-age birth weight (customized centile <10).

**Figure 2.**
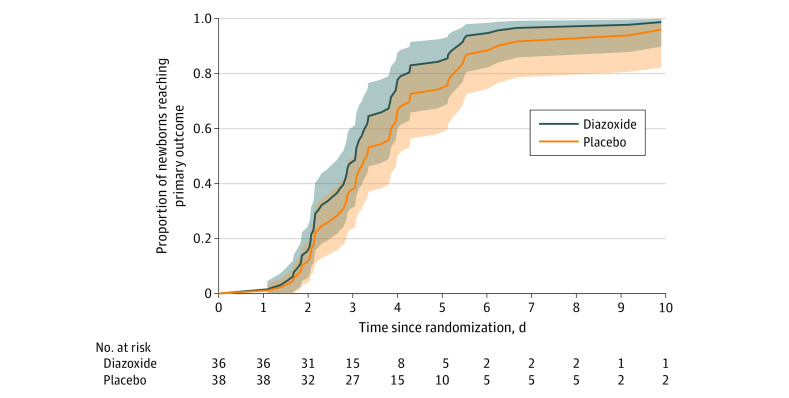
Time to Resolution of Hypoglycemia Estimated survival functions were adjusted for stratification variables (site and customized birth weight centile) and gestation length. Shading indicates 95% CIs. Time to resolution of hypoglycemia was defined as the point at which all of the following occurred concurrently for at least 24 hours: enteral bolus feeding; normoglycemia (all blood glucose concentrations within the target range of 47-98 mg/dL; to convert to mmol/L, multiply by 0.0555); and no intravenous fluids.^8^ Two newborns in the diazoxide group did not reach the primary outcome, and their data were censored: 1 at 8.65 days due to persisting mildly elevated blood glucose concentration (99-104 mg/dL>5 days after ceasing the intervention) and 1 at 2.01 days due to being transferred to a nonparticipating center. One newborn in the placebo group did not reach the primary outcome and was censored at 19.21 days due to commencing open-label diazoxide treatment (intervention discontinued) and the responsible endocrinologist electing to target a higher blood glucose concentration range. Two other newborns in the placebo group began open-label diazoxide treatment, as hypoglycemia could not be adequately controlled (intervention discontinued); both these newborns reached the primary outcome and were included in the analysis without censoring. In all cases, investigators and research personnel remained blinded to group allocation until data lock and analysis.

There was no significant difference in time to resolution of hypoglycemia between the intervention and placebo groups (AHR, 1.39; 95% CI, 0.84-2.23) (Figure 2). However, the diazoxide group compared with the placebo group had reduced time to bolus feeding (ARGM, 0.74 [95% CI, 0.58-0.95]), duration of intravenous fluids (ARGM, 0.72 [95% CI, 0.60-0.87]), number of episodes of hypoglycemia (ACR, 0.32 [95% CI, 0.17-0.63]), duration of hypoglycemia (ARGM, 0.18 [95% CI, 0.06-0.53]), and number of blood glucose tests (ACR, 0.63 [95% CI, 0.56-0.71]) (Table 2). There was no difference in the duration of admission between groups (ARGM, 0.85 [95% CI, 0.66-1.09]) (Table 2). Eleven newborns (29%) in the placebo group had the study drug increased by protocol because of ongoing hypoglycemia (along with increases in fluids and/or feedings) (eFigure 1 in Supplement 2), but this was required for only 2 newborns (6%) receiving the diazoxide intervention (Table 2). Only 2 newborns (6%) in the diazoxide group had any hypoglycemia after completing the loading dose compared with 20 (53%) in the placebo group.

**Table 2.** Secondary Outcomes

Outcome	Diazoxide group		Placebo group		Adjusted effect size (95% CI)^b^
	Value^a^	Total No.	Value^a^	Total No.	
Time to achieve normoglycemia, median (IQR), d^c^	1.96 (1.14-2.87)	34	1.16 (1.04-1.93)	37	1.29 (1.00-1.67)^d^
Time to establish enteral bolus feeding, median (IQR), d	1.75 (1.00-2.43)	36	2.11 (1.58-3.15)	37	0.74 (0.58-0.95)^d^
Time to establish full sucking feeding, median (IQR), d^e^	3.88 (1.86-6.70)	35	5.38 (2.76-8.09)	38	0.94 (0.61-1.48)^d^
Exclusive breastfeeding from birth	0	36	4 (11)	38	NA
Full breastmilk feedings at discharge at 48 h	21 (58)	36	18 (47)	38	1.42 (0.55-3.68)^f^
Use of intravenous dextrose	32 (89)	36	36 (95)	38	0.49 (0.08-2.91)^f^
Dextrose concentration ≥15%	2 (6)	36	7 (18)	38	0.30 (0.05-1.61)^f^
Duration of intravenous dextrose, median (IQR), d	2.30 (1.83-2.92)	32	3.31 (2.56-4.28)	36	0.72 (0.60-0.87)^d^
Hypoglycemia^c^					
Episodes, median (IQR), No.	0 (0-0)^g^	36	1 (0-2)^h^	38	0.32 (0.17-0.63)^i^
Total duration of episodes, median (IQR), h^j^	0.0 (0.0-0.0)	36	0.8 (0.0-2.9)	38	0.18 (0.06-0.53)^d^
Hypoglycemia after achieving normoglycemia^c^	1 (3)	35	6 (16)	38	0.17 (0.02-1.57)^f^
Hypoglycemia >48 h after randomization^c^	1 (3)	36	5 (13)	38	0.19 (0.02-1.76)^f^
Study drug increased for hypoglycemia^k^	2 (6)	36	11 (29)	38	0.14 (0.03-0.70)^f^
Open-label diazoxide due to inability to achieve blood glucose stability	0	36	3 (8)	38	NA
Hypoglycemia after stopping study drug	0	36	0	38	NA
Blood glucose tests, median IQR, No.					
During study intervention	11 (9-13)	36	18 (13-21)	38	0.63 (0.56-0.71)^i^
Up to hospital discharge	15 (12-20)	36	20 (15-28)	38	0.67 (0.60-0.75)^i^
Time to neonatal unit discharge, median (IQR), d	2.78 (1.78-4.55)	36	3.38 (2.27-6.80)	38	0.75 (0.50-1.12)^d^
Time to discharge to home, median (IQR), d	6.05 (3.92-7.97)	36	7.50 (4.96-10.92)	38	0.85 (0.66-1.09)^d^
Commencement of low-flow oxygen or positive pressure respiratory support	3 (8)	36	4 (11)	38	0.65 (0.13-3.32)^f^
Congestive heart failure	0	36	0	38	NA
Elevated blood glucose concentration^c^					
Episodes, median (IQR), No.	2 (1-3)	36	0 (0-1)	38	2.65 (1.72-4.11)^i^
Total duration of episodes, median (IQR), h^j^	10.7 (4.5-23.1)	36	0.0 (0.0-5.9)	38	25.21 (6.76-94.05)^d^
Elevated blood glucose concentration >48 h after randomization	17 (47)	36	7 (18)	38	4.10 (1.39-12.18)^f^
Study drug stopped for elevated glucose concentration	8 (22)	36	2 (5)	38	7.34 (1.10-49.29)^f^
Hyperglycemia^c^					
Episodes, median (IQR), No.	0 (0-1)	36	0 (0-0)	38	3.04 (1.24-7.45)^i^
Total duration of episodes, median (IQR), h^j^	0.0 (0.0-6.1)	36	0.0 (0.0-0.0)	38	3.87 (1.04-14.39)^d^
Hyperglycemia >48 h after randomization	4 (11)	36	3 (8)	38	1.43 (0.29-7.13)^f^
Study drug stopped for hyperglycemia	0	36	0	38	NA
Plasma insulin concentration 36 h after intervention, median (IQR), μIU/mL	0.4 (0.2-0.9)	29	0.8 (0.5-1.3)	29	0.50 (0.32-0.79)^d^
Plasma creatinine concentration 36 h after intervention, mean (SD), mg/dL	0.5 (0.2)	28	0.5 (0.2)	27	4.0 (−3.1-11.1)^l^
Duration of study intervention, median (IQR), d	2.48 (1.94-3.24)	36	3.90 (2.98-5.54)	38	0.66 (0.54-0.80)^d^

Abbreviation: NA, not applicable.

SI conversion factors: To convert blood glucose concentration to mmol/L, multiply by 0.0555; creatinine to μmol/L, multiply by 88.4; insulin to pmol/L, multiply by 6.945.

^a^ Data are presented as number (percentage) of neonates unless otherwise indicated.

^b^ Generalized linear models, adjusted for stratification variables (site and customized birth weight centile) and gestation length.

^c^ Normoglycemia was defined as blood glucose concentration of 47 to 98 mg/dL for 24 hours; hypoglycemia, less than 47 mg/dL; elevated blood glucose, 99 to 124 mg/dL; and hyperglycemia, 125 mg/dL or greater.

^d^ Ratio of geometric mean.

^e^ Full sucking feedings were defined as 5 or more full breast feedings (≥10 minutes) in 24 hours or at least 120 mL/kg/d of expressed breast milk or formula by bottle; all newborns achieved full sucking feedings before discharge to home.

^f^ Odds ratio.

^g^ In the diazoxide group, 3 neonates had a single episode of mild hypoglycemia (blood glucose level of 36.0-45.0 mg/dL), but in 1 case the episode was less than 30 minutes after the first dose; 1 newborn had a severe (<36.0 mg/d) episode, but the severe episode was less than 30 minutes after the first dose.

^h^ In the placebo group, 12 neonates had a single mild episode, 6 had 2 mild episodes, 1 had 4 mild episodes, and 1 had a mild and a severe episode.

^i^ Count ratio.

^j^ Sum of episodes.

^k^ Three newborns in the placebo group received open-label diazoxide due to persisting hypoglycemia.

^l^ Mean difference.

Despite these effects, the time to achievement of normoglycemia (blood glucose concentration of 47-98 mg/dL) for at least 24 hours appeared to be longer in the diazoxide group (ARGM, 1.29 [95% CI, 1.00-1.67]) (Table 2). This was attributable to more episodes of elevated blood glucose concentration (99-124 mg/dL) (diazoxide: median, 2 [IQR, 1-3]; placebo: median, 0 [IQR, 0-1]), although this was relatively frequent in both groups (ACR, 2.65 [95% CI, 1.72-4.11]) (Table 2). Hyperglycemia (blood glucose concentration ≥125 mg/dL) also occurred in both groups but was infrequent; no newborns had the intervention stopped because of hyperglycemia (Table 2).

There was no evidence that diazoxide was associated with increased use of oxygen or respiratory support or with congestive heart failure (Table 2). Among newborns who underwent cardiac ultrasonography (diazoxide: n = 29; placebo: n = 33), 1 (3%) in the diazoxide group had a patent ductus arteriosus that closed spontaneously within 48 hours; none had pulmonary hypertension or cardiac impairment by predefined criteria.^8^ One newborn (3%) in the diazoxide group had a seizure 2 days after randomization, but the blood glucose concentration was normal and brain imaging demonstrated a neuronal migration disorder; the data safety and monitoring committee judged the seizure to be unrelated to the trial. No newborn developed gastrointestinal bleeding, feeding intolerance, or necrotizing enterocolitis. All newborns survived to hospital discharge and had a normal newborn metabolic screening result. There were no cases of congenital hyperinsulinism, and no newborns were readmitted for hypoglycemia after discharge. All newborns had the intervention or open-label diazoxide stopped before discharge from the hospital.

At 36 hours after the loading dose, median insulin concentrations were reduced by 50% in the diazoxide group compared with the placebo group, but plasma creatinine concentrations were similar between groups (Table 2). The mean (SD) plasma diazoxide concentration in the active intervention group was 20.4 (5.3) μg/mL (n = 28).

### Post Hoc Analyses

When resolution of hypoglycemia was redefined as enteral bolus feeding without intravenous fluids for at least 24 hours with no further hypoglycemia, the rate of attainment was higher with the diazoxide intervention (AHR, 2.60 [95% CI, 1.53-4.46]; Hodges-Lehmann estimation of median time, 2.2 days [95% CI, 1.9-2.8 days] in the diazoxide group and 3.3 days [95% CI, 2.8-3.91-4.3 days] in the placebo group) (eFigure 2 in Supplement 2). There was a high insulin-to-glucose ratio in both groups at baseline (diazoxide: median, 4.5 U/mol [IQR, 2.0-7.9 U/mol]; placebo: median, 5.2 U/mol [IQR, 2.7-10.8 U/mol]). The median insulin-to-glucose ratio at 36 hours after the loading dose was reduced with diazoxide (14 [39%]) compared with placebo (14 [37%]) among newborns with paired insulin and glucose data included (diazoxide: median, 0.5 U/mol [IQR, 0.2-1.2 U/mol]; placebo: median, 1.3 U/mol [IQR, 1.0-2.7 U/mol]; ARGM, 0.32 [95% CI, 0.15-0.65]) (eFigure 3 in Supplement 2). Group mean interstitial glucose concentrations started to differ at 1.5 hours after the loading dose (eFigure 4 in Supplement 2). After the last intervention dose, mean interstitial glucose concentrations remained at 81 to 90 mg/dL in the diazoxide group (the normal level for newborns who have completed metabolic transition^14,16^), whereas interstitial glucose concentrations continued to increase in the placebo group for several days (eFigure 5 in Supplement 2). Many newborns in this group continued to have hypoglycemia more than 48 hours after trial entry (5 [13%]) and after initial stabilization of blood glucose concentration (6 [16%]). In a sensitivity analysis that included only newborns born small or large for gestational age (customized birth weight centile <10 or >90, respectively), the difference in interstitial glucose concentrations between groups was similar to that in the whole cohort (eFigures 6 and 7 and eTables 1 and 2 in Supplement 2). In the diazoxide group, trough plasma diazoxide concentrations were not correlated with mean interstitial glucose concentrations at 12 or more hours (Pearson *r*, −0.22; *P* = .33).

## Discussion

We found no evidence that diazoxide reduced time to resolution of hypoglycemia as defined as the primary outcome. However, there was a clinically important reduction in the time to achieve enteral bolus feeding and weaning from intravenous dextrose, both components of the primary outcome, as well as reduced heel pricks for blood glucose testing. In the post hoc analysis, when resolution of hypoglycemia was redefined as enteral bolus feeding without intravenous fluids for at least 24 hours with no further hypoglycemia, there was a 2-fold increase in the rate at which this end point was reached in the diazoxide group compared with the placebo group.

We took a cautious approach in defining the primary outcome, as animal studies^17,18^ and indirect evidence from humans^5,19^ have suggested that rapid correction of hypoglycemia or overcorrection to too high a concentration may exacerbate oxidative injury in the developing brain, a phenomenon termed *glucose reperfusion injury*.^18^ Thus, we defined normoglycemia as maintaining a blood glucose concentration within the 80% central range (10th-90th centiles) reported for healthy full-term breastfed neonates in the first week after birth.^14^ However, it remains unclear whether this target is appropriate for neonates receiving treatment for hypoglycemia, and the extent to which glucose reperfusion injury occurs in human neonates and under what conditions requires further confirmation in clinical trials and follow-up studies. While more neonates in the diazoxide group experienced a blood glucose concentration above the 90th centile (98 mg/dL), true hyperglycemia (≥125 mg/dL) was rare.

Of concern is the high ongoing burden of hypoglycemia in the placebo group despite these newborns being admitted to neonatal care and receiving close monitoring and standard titration of intravenous dextrose and feedings. Many of these newborns continued to have hypoglycemia more than 48 hours after trial entry (13%) and after initial stabilization of blood glucose concentration (16%). Despite its widespread use, treatment of neonatal hypoglycemia with intravenous dextrose has not been evaluated in clinical trials to our knowledge, and data on its effectiveness are limited to a small case series spanning not more than a few hours.^20^ In contrast, in this trial, hypoglycemia was almost eliminated by treatment with low-dose diazoxide, with only 2 newborns (6%) having a single brief episode after completion of the loading dose. This likely contributed to the reduced number of heel pricks that newborns in the diazoxide group received.

The 2 newborns in the diazoxide group who experienced hypoglycemia after the loading dose required only a single increase in the study drug regimen. Thus, it is possible that a lower starting maintenance dose may be similarly effective in correcting hypoglycemia while avoiding elevated blood glucose concentration. Alternatively, diazoxide could be used instead of intravenous dextrose, and the dose used in this trial may prove to be sufficient. Diazoxide could also be used in place of formula supplementation, which was almost universal in this population despite most mothers wanting to breastfeed. Immediate use of diazoxide when dextrose gel and breastfeeding fail to stabilize blood glucose concentration warrants further investigation, including feasibility in nontertiary care environments. Diazoxide has many advantages that make it suitable as a second-line agent, including low cost (approximately $12 per newborn with local compounding^13^), oral administration, twice-daily dosing, and dose responsiveness.^9^

One previous trial investigated the use of high-dose diazoxide (9-12 mg/kg/d) to treat neonatal hypoglycemia in newborns with low birth weight (<2.5 kg) in the first week after birth.^9^ Similar to our trial, Balachandran et al^15^ found that high-dose diazoxide reduced the duration of intravenous dextrose and time to achieve full enteral feeding by approximately 2 days. The time to achieve normoglycemia was also reduced, but in contrast to our primary outcome, this was defined by correction of hypoglycemia without consideration of an elevated blood glucose concentration. Our starting dose was only one-third of the dose used by Balachandran et al,^15^ so it is possible that hyperglycemia occurred in that study even though it was not reported. Balachandran et al^15^ restricted their trial to newborns with low birth weight, but our trial showed that diazoxide may be applicable to a wider population of at-risk newborns, including those born large for gestational age, and is feasible to use even on the first postnatal day. Despite the encouraging results of these 2 trials, replication in other care environments will be important, including where different operational thresholds for neonatal hypoglycemia apply.

Neonatal hypoglycemia is often described as a state of hyperinsulinism, but baseline insulin concentrations were not high in this study, as our group has observed previously.^7^ Nevertheless, at baseline, there was a high insulin-to-glucose ratio in both groups, suggesting that the primary defect in these newborns was a failure to adequately suppress insulin secretion at a low blood glucose concentration. Recent evidence has pointed to lower K_ATP_ trafficking, reducing membrane polarity, as contributing to a low glucose set point for insulin secretion in immature β cells.^21^ Amino acid–mediated insulin secretion is also important in the fetus and must be downregulated after birth.^22,23^ Interestingly, in a previous cohort of newborns with persistent hypoglycemia, our group observed a similar insulin-to-glucose ratio,^7^ indicating that hypoglycemia risk after birth may be related to the time required for maturation of β-cell signaling and membrane function rather than the magnitude of insulin secretion. We speculated that diazoxide would shift this glucose set point in a dose-dependent manner.^9^ In support of this, diazoxide halved insulin concentrations at 36 hours compared with placebo, but it had an even larger effect on the insulin-to-glucose ratio (68% reduction compared with placebo). We did not observe rebound hypoglycemia after stopping diazoxide, suggesting that low-dose diazoxide therapy may support β-cell adaptation, although further mechanistic studies are required.^21,23,24^

Cardiac myocytes, vascular smooth muscle cells, and endothelial cells also contain K_ATP_,^25^ and there have been sporadic reports of high-dose diazoxide being associated with reversible congestive heart failure, edema, patent ductus arteriosus, and pulmonary hypertension,^26,27^ which may be reduced with concomitant use of diuretics^26^; however, we did not observe any such effects, even in newborns systematically screened by routine cardiac ultrasonography. Similarly, diazoxide use in neonates has been associated with necrotizing enterocolitis,^28^ but there were no gastrointestinal complications in our trial or in the newborns with low birth weight studied by Balachandran et al.^15^ Thus, low-dose diazoxide may be well tolerated in otherwise healthy late preterm through full-term newborns who have not been exposed to perinatal asphyxia and do not have any underlying cardiovascular, kidney, or gastrointestinal disorders. However, this study had limited power to detect potential adverse effects, and further randomized data on safety are needed.

There are few pharmacokinetic data for oral diazoxide dosage in neonates.^26^ We used the lowest daily maintenance dose recommended in national guidelines,^29^ divided every 12 hours, as a previous simulation study in infants showed that 12- and 8-hourly dosing achieved comparable steady state concentrations.^12^ Trough plasma diazoxide concentrations did not appear to be related to glucose concentrations, suggesting that at low doses, factors related to β-cell maturation may be more important than pharmacokinetics in determining clinical response.

### Strengths and Limitations

The strengths our of trial include effective blinding, formulation of diazoxide in suspension at a concentration that is practical to titrate, enrollment of neonates soon after the onset of hypoglycemia, measurement of all blood glucose concentrations by gas analyzer, use of standardized processes to implement the intervention, and use of routine cardiac ultrasonography assessment at the main site. The limitations include a modest sample size and risk of type II error for smaller clinical differences and infrequent outcomes, a relatively short period of observation after discontinuation of the intervention, and high overall use of formula, which may have obscured potential benefits of breastfeeding.

## Conclusions

In this randomized clinical trial, in late preterm through full-term newborns with severe or recurrent hypoglycemia, early low-dose oral diazoxide did not reduce time to resolution of hypoglycemia but reduced time to establishing enteral bolus feeding and weaning from intravenous fluids and reduced the duration of hypoglycemia and frequency of heel pricks for blood glucose testing compared with placebo. Further investigation of the use of diazoxide is warranted in newborns whose condition is refractory to initial management of transitional hypoglycemia with dextrose gel and breastfeeding.

## Notes

Supplement 1.
Trial Protocol

Supplement 2. **eFigure 1.** Intravenous Dextrose Delivery After Commencing Intervention **eFigure 2.** Resolution of Hypoglycemia, Post Hoc Definition **eFigure 3.** Insulin to Glucose Ratio at 36 Hours After Commencing Intervention **eFigure 4.** Interstitial Glucose After Last Intervention Dose **eFigure 5.** Interstitial Glucose Immediately After Intervention Loading Dose **eFigure 6.** Interstitial Glucose From Commencement of Intervention **eTable 1.** Estimated Difference in Interstitial Glucose Between Diazoxide vs Placebo Groups **eFigure 7.** Interstitial Glucose Among Neonates Born Small or Large for Gestational Age From Commencement of Intervention **eTable 2.** Estimated Difference in Interstitial Glucose Between Diazoxide vs Placebo Groups Among Neonates Born Small or Large for Gestational Age

Supplement 3.
Data Sharing Statement
