# Supplementary material for: Diazoxide for Severe or Recurrent Neonatal Hypoglycemia: A Randomized Clinical Trial
Source: JAMA Netw Open. 2024 Jun 13;7(6):e2415764. doi: 10.1001/jamanetworkopen.2024.15764 (PMC11177163; doi:10.1001/jamanetworkopen.2024.15764)
Supplement: Supplement 1. — Trial Protocol [file jamanetwopen-e2415764-s001.pdf]

STUDY PROTOCOL

NEO GLU C O
Neonatal Glucose Care Optimisation Study

Oral diazoxide versus placebo for severe or recurrent neonatal hypoglycaemia

The Neonatal Glucose Care Optimisation (NeoGluCO) Study

STUDY PROTOCOL

ADMINISTRATIVE INFORMATION

Oral diazoxide versus placebo to reduce time to successful treatment of hypoglycaemia in neonates with severe or recurrent hypoglycaemia: The Neonatal Glucose Care Optimisation (NeoGluCO) Study.

Short title: NeoGluCO Study

Registration: universal trial number U1111-1242-9558; ANZCTR12620000129987

Protocol details: version 2.6, date 2022.6.27

Funding: University of Auckland, Health Research Council

Host Organisation (Sponsor): University of Auckland

Coordinating Investigator:

Chris McKinlay, Paediatrics: Child Youth Health, University of Auckland
Private Bag 92019, Auckland 1142, New Zealand
Email: c.mckinlay@auckland.ac.nz
Phone: +64 274725099

Investigators:

Jane Alsweiler, Paediatrics: Child Youth Health, University of Auckland
Jane Harding, Liggins Institute, University of Auckland
Wayne Cutfield, Liggins Institute, University of Auckland
Don Laing, Liggins Institute, University of Auckland
Jenny Rogers, Liggins Institute, University of Auckland
Greg Gamble, Liggins Institute, University of Auckland
Geoff Chase, College of Engineering, University of Canterbury
Sara Hanning, School of Pharmacy, University of Auckland
Julena Ardern, Kidz First Neonatal Care, Counties Manukau Health
Mike Meyer, Kidz First Neonatal Care, Counties Manukau Health

Study Sites:

Kidz First Neonatal Care, Middlemore Hospital, Counties Manukau Health (CM Health), Auckland

Neonatal Intensive Care Unit, Auckland City Hospital, Auckland District Health Board (ADHB)

Roles and responsibilities:

Trial Steering Committee: Investigators

Site Lead Investigators: Chris McKinlay (CM Health), Jane Alsweiler (ADHB)

Study Coordinator: Lisa Mravicich, Research Midwife, Liggins Institute, University of Auckland

Management Committee: Chris McKinlay, Jane Alsweiler, Lisa Mravicich, Don Laing, Eamon Walsh, Julena Ardern, Sabine Huth (Research Nurse)

Data Management: Liggins Institute

Data Monitoring and Safety Committee: Prof. Stuart Dalziel (Chair), Prof. Rinki Murphy, A/Prof. Nicola Austin

This study protocol follows the SPIRIT checklist.[1,2]

Contents

1 INTRODUCTION 5
1.1 Background and Rationale 5
1.2 Objectives and Hypotheses 7
1.3 Study Design and Synopsis 7
2 METHODS 8
2.1 Participants, Interventions and Outcomes 8
2.1.1 Study Setting 8
2.1.2 Eligibility Criteria 8
2.1.3 Interventions 9
2.1.4 Glucose and fluid management after randomisation 9
2.1.5 Titration of the intervention 10
2.1.6 Continuous glucose monitoring 12
2.1.7 Other co-interventions 12
2.1.8 Study Assessments 12
2.1.9 Outcomes 13
2.1.10 Participant Timeline 14
2.1.11 Sample Size and Feasibility 15
2.1.12 Recruitment 15
2.2 Assignment of Interventions 15
2.2.1 Allocation Sequence Generation 15
2.2.2 Allocation Concealment Mechanism 15
2.2.3 Implementation 15
2.2.4 Blinding (Masking) 15
2.2.5 Emergency Unblinding 15
2.3 Data Collection, Management and Analysis 16
2.3.1 Data Collection Methods 16
2.3.2 Retention 16
2.3.3 Data Management 16
2.3.4 Statistical Methods 16
2.4 Monitoring 17
2.4.1 Data Monitoring and Safety Committee 17
2.4.2 Interim Analysis 17
2.4.3 Harms 17
3 ETHICS AND DISSEMINATION 17
3.1 Research Ethics Approval 17
3.2 Locality Approval 17

3.3 Protocol Amendments 18
3.4 Consent 18
3.5 Withdrawal 18
3.6 Ancillary Studies 18
3.7 Confidentiality 18
3.8 Declaration of Interests 18
3.9 Access to Data 19
3.10 Dissemination Policy 19
3.11 Authorship policy 19
3.12 Data Sharing Policy 19
3.13 Māori Responsiveness 19
4 STUDY MANAGEMENT 19
4.1 Steering Committee 19
4.2 Management Committee 19
4.3 Site Principal Investigator 19
4.4 Finance and Insurance 20
5 APPENDICES 21
5.1 Protocol Amendments 21
5.2 Participant Documents 21
5.3 Ethical and Locality Approval 22
5.4 Study Committees 22
5.5 Standard Operating Procedures 22
5.6 Funding 22
6 REFERENCES 23

1 INTRODUCTION

1.1 Background and Rationale

Neonatal hypoglycaemia is a significant clinical problem with the potential for long-term sequelae

At least 30% of all newborn babies or 20,000 per annum in New Zealand (NZ) are at risk of transitional hypoglycaemia (low blood glucose concentration, BGC) due to being born small, large, preterm or the infant of a diabetic mother. They require regular testing of BGC in the first 24 to 48 hours after b

irth and approximately 50% develop hypoglycaemia and require further testing and intervention.[3] Optimal management of transitional neonatal hypoglycaemia is important not only because of its impact on breastfeeding[4,5] and use of health care resources,[6,7] but also because of the potential for permanent brain injury. The immature brain is dependent on a continuous supply of glucose for energy, as other brain fuels such as ketones are low after birth.[8,9] We have shown that babies with asymptomatic hypoglycaemia have a two- to three-fold increased likelihood of later neurocognitive difficulties by 4 to 5 years of age, especially of executive function and visual-motor integration.[10-12] These functions are critical for learning, and even brief transitional neonatal hypoglycaemia has been associated with a two-fold increased likelihood of poor school achievement.[13] In moderately preterm infants, transitional hypoglycaemia is the main modifiable risk factor for developmental delay at preschool age.[14]

Better treatment is needed for babies with severe or recurrent neonatal hypoglycaemia

If oral dextrose gel and additional feeding do not correct hypoglycaemia, babies are typically admitted to the neonatal unit for frequent or continuous feeding by gastric tube or intravenous glucose, with the aim of correcting glucose concentrations as rapidly as possible to a high normal level.[3,15] Approximately 20% of babies with neonatal hypoglycaemia require neonatal admission for severe or recurrent hypoglycaemia (1,800 per annum in NZ).[15] These babies often have prolonged neonatal admission, ongoing hypoglycaemia despite the provision of intravenous fluids, and can be difficult to establish on enteral feeds due to glucose instability. Even with standard management, babies with severe or recurrent transitional hypoglycaemia continue to have substantially higher rates (approximately four-fold) of adverse neurological outcome.[11] This may be due, at least in part, to additional oxidative injury from too rapid correction of hypoglycaemia,[16,17] and the fact that episodes of clinically undetected hypoglycaemia are common in these babies, further increasing the risk of brain injury.[11] Thus, the current treatment of babies with severe or recurrent transitional hypoglycaemia remains sub-optimal and better management strategies are needed that address the underlying pathophysiology.

Effective management strategies are needed that target the underlying pathophysiology

The primary cause of severe or recurrent transitional neonatal hypoglycaemia is dysregulated insulin secretion, especially the inability to suppress insulin secretion at low BGCs and with fasting. During fetal life, insulin is a key growth hormone that is secreted by the fetal pancreas in response to placental uptake of glucose and free fatty acids, with a low set-point for insulin release.[9] Fetal glucose synthesis is negligible because the continuous supply of glucose via the placenta stimulates fetal insulin secretion. Hepatic expression of enzymes for gluconeogenesis and glycogenolysis is also low until late gestation. At birth, placental glucose supply ceases and the neonate must adapt to bolus feeding and intermittent fasting. Successful neonatal metabolic transition requires the initiation of glycogenolysis (release of glucose from hepatic glycogen), gluconeogenesis (synthesis of glucose in the liver) and lipolysis (release of fuels from fat, including glycerol for glucose synthesis), all of which are inhibited by insulin. If insulin secretion remains inappropriately high during this transition period, hepatic glucose output is inadequate for metabolic requirements, and hypoglycaemia ensues. Increasing delivery of exogenous glucose, either with formula or intravenous

dextrose, may result in a vicious cycle of further insulin secretion and ongoing hypoglycaemia, despite the escalating provision of glucose. Further, the counter-regulatory mechanisms of glucagon, cortisol and growth hormone, which promote glycogenolysis (release of hepatic glucose stores) and gluconeogenesis, may be less effective in neonates with hyperinsulinaemic hypoglycaemia,[18,19] and coupling of cerebral blood flow and metabolic demand may also be perturbed.[20,21]

Under normal conditions, the brain accounts for the majority neonatal glucose utilisation,[22,23] and uptake is by facilitated diffusion, independent of insulin.[24] Thus, higher circulating concentrations of insulin during neonatal transition, rather than facilitating cerebral energy uptake, increase the risk of neuroglycopenia by limiting hepatic glucose output. Further, the capacity for cerebral glucose uptake after birth may be rate-limited as the maximal expression of glucose transporter proteins at the blood-brain barrier (GLUT1 and GLUT3) does not occur for several days to weeks.[24] Ketone bodies are an important cerebral alternative fuel, but higher circulating insulin concentrations also suppress lipolysis (releases free fatty acids) and hepatic beta-oxidation, such that ketones are largely absent in babies with hypoglycaemia.[8,25]

Both large and small babies are at risk of dysregulated insulin secretion. Babies who are large for gestation are typically born to mothers with obesity or diabetes (pre-existing or gestational), which can lead to excess fetal supply of glucose and free fatty acids. This increases fetal insulin secretion, which in turn increases fetal pancreatic beta cell mass (hypertrophy and hyperplasia), adaptations that persist for a period after birth.[26,27] Conversely, in growth restriction and placental insufficiency, fetal supply of oxygen and nutrients is reduced. Fetal hypoxaemia raises plasma catecholamine concentrations, especially noradrenaline, which acts on beta cells to suppress *in utero* insulin secretion.[28,29] At birth, loss of the sustained adrenergic signalling exposes beta cell hyper-responsiveness, resulting in increased insulin secretion. Thus, both fetal over- and under-nutrition can result in transient neonatal hyperinsulinism and associated hypoglycaemia.

Achieving glucose stability in neonates with severe or recurrent hypoglycaemia is challenging not only because of an exaggerated glucose-stimulated insulin response but also because with escalating treatment, episodes of hyperglycaemia also occur. This risk is greatest in fetal growth restriction and with the use of intravenous dextrose.[16] The reasons for this instability are not fully known, but it may be due to periodic impairment of peripheral glucose disposal due to variations in insulin sensitivity or temporary depletion of beta cell insulin vesicles. Importantly, we have shown that higher glucose concentrations after hypoglycaemia or correction that is too rapid may exacerbate brain injury,[12,16] as has been demonstrated in animals.[30,31] This suggests that the goal of management should be glucose stability rather than simply correction of hypoglycaemia per se. To achieve optimal glycaemic control in neonates with severe or recurrent transitional hypoglycaemia new treatment approaches are needed that target the underlying pathophysiology, namely, dysregulation of insulin secretion.

Diazoxide is a potential new management strategy to improve treatment of neonatal hypoglycaemia

Diazoxide acts on the pancreatic beta cell in a dose-dependent manner to decrease insulin secretion by interacting with the sulfonylurea receptor (SUR1). Binding of diazoxide to the SUR1 subunit inhibits closure of the ATP-sensitive potassium (K) channel, which in turn diminishes first and second phase glucose-stimulated insulin secretion.[32-34] Advantages of diazoxide include rapid onset of action, oral formulation and low cost. Diazoxide has been used for many decades as first-line treatment for certain forms of congenital (genetic) hyperinsulinism, with a good efficacy and safety profile.[35]

It has also been used selectively in babies with transient hyperinsulinism. Hoe *et al.* described 21 hyperinsulinaemic babies without known genetic defect, 20 (95%) of whom were responsive to diazoxide (5 to 10 mg/kg/day), when commenced at a median age of 13 days. In a retrospective case series, we identified eight late preterm or term neonates with severe or recurrent transitional

hypoglycaemia who were commenced on diazoxide in the first week (unpublished data). Diazoxide was effective in facilitating weaning of intravenous dextrose and transition to enteral feeds, although six neonates had episodes of hyperglycaemia ≥7 mmol/L, most likely due to use of maintenance doses that were potentially too high (5 to 10 mg/kg/day) and not adequately weaned.

In a small randomised trial of 30 small-for-gestational age neonates with transient hyperinsulinism in the first five days, diazoxide at 6 to 12 mg/kg/day reduced the median time to achieve hypoglycaemic control (40 vs 72 hours, P=0.02), the total duration of intravenous fluids (114 vs 164 hours, P=0.04) and time to achieve full feeds (74 vs 124 hours, P=0.02).[36] There were no apparent adverse events, although episodes of hyperglycaemia were not reported.

In babies with congenital hyperinsulinism on regular diazoxide, cardiac complications have been rarely reported, including congestive heart failure, patent ductus arteriosus and pulmonary hypertension.[37,38] In all cases, there was full resolution of symptoms on stopping diazoxide. Similarly, in children recently diagnosed with type 1 diabetes and treated with regular diazoxide at 5-7.5 mg/kg/day, 5% experienced reversible oedema, but no serious adverse effects were reported.[39]

Together these data suggest that diazoxide may have a role in early management of severe neonatal hypoglycaemia to reduce the need for intravenous glucose, shorten neonatal unit admissions and facilitate earlier introduction of enteral feeds, and that low dose that treatment is likely to be well tolerated.

The NeoGluCO Study

We propose the Neonatal Glucose Care Optimisation (NeoGluCO) Study to investigate if early use of oral diazoxide in severe or recurrent neonatal hypoglycaemia results in the earlier establishment of enteral bolus feeding and normal glucose concentrations without intravenous fluids. If effective, such a treatment could have major benefits for neonates with severe or recurrent hypoglycaemia, including reduced length of admission and separation of mother and baby, reduced use of formula and facilitation of the earlier establishment of breastfeeding, reduced number of heel pricks for BGC testing, and better long-term neurodevelopmental outcomes.

1.2 Objectives and Hypotheses

Primary objective: To determine if early use of diazoxide in severe or recurrent neonatal hypoglycaemia reduces time to resolution of hypoglycaemia, defined as achieving enteral bolus feeding and normal glucose concentrations without intravenous fluids (see below).

Primary hypothesis: Early diazoxide therapy will improve glycaemic stability, allowing earlier weaning of intravenous fluids and establishment of enteral feeds.

1.3 Study Design and Synopsis

Phase 2, Double-blinded, randomised controlled, two-arm, parallel trial of diazoxide versus placebo in neonates born at ≥35 weeks' gestation admitted to neonatal care with severe or recurrent hypoglycaemia in the first week (Table 1). Severe hypoglycaemia is defined as any BGC <1.2 mmol/L or BGC 1.2 to <2.0 mmol/L despite two doses of dextrose gel and feeding in a single episode; recurrent hypoglycaemia is defined as ≥3 episodes (one or more consecutive BGCs) of hypoglycaemia <2.6 mmol/L in 48 h. Babies will be randomised to either oral diazoxide 5 mg/kg load, then 1.5 mg/kg 12 hourly or an equivalent volume of placebo. Management of feeds, fluid and hypoglycaemic episodes will occur according to local practice. Once glycaemic stability is achieved, the study drug will be weaned by protocol. If hyperglycaemia occurs (≥7.0 mmol/L), the study drug will be discontinued. The primary outcome is time to resolution of hypoglycaemia, defined as achieving enteral bolus feeding and normal glucose concentrations without intravenous fluids (see below).

Table 1: PICOT Summary

**Participants**	Neonates ≥35 weeks admitted to the neonatal unit with severe or recurrent hypoglycaemia in the first week.
**Intervention**	Oral diazoxide 5 mg/kg loading dose, then 1.5 mg/kg 12 hourly maintenance dose, weaning by protocol.
**Control**	An equivalent volume of identical placebo.
**Primary outcome**	Time to resolution of hypoglycaemia, defined as achieving enteral bolus feeding and normal glucose concentrations without intravenous fluids.
**Planned sample size**	74 babies will be randomised in 1:1 ratio, giving 80% power to detect a relative hazard of 2.0 (2-tailed alpha 0.05).
**Timing of assessment**	Assessment for the primary outcome will continue for up to 4 weeks, after which the primary outcome will be censored.

2 METHODS

2.1 Participants, Interventions and Outcomes

2.1.1 Study Setting

Counties and Auckland DHB Neonatal Care Units.

2.1.2 Eligibility Criteria

Babies are eligible for this study if they are born at ≥35 weeks and are admitted to a neonatal unit in the first week after birth with recurrent or severe hypoglycaemia, defined by one or more of the following:

- Any episode of hypoglycaemia <1.2 mmol/L
- BGC of 1.2 to <2.0 mmol/L persisting after 2 doses of dextrose gel and feeding in a single episode
- ≥3 episodes of hypoglycaemia <2.6 mmol/L in 48 h

Babies must also be receiving ongoing management for hypoglycaemia at the time of randomisation, e.g., intravenous dextrose, carbohydrate supplements, continuous or frequent feeding (≤2 hourly), or inability to wean off formula due to hypoglycaemia.

The following babies will be excluded from randomisation:

- Confirmed major congenital malformation or chromosomal disorder
- Suspected genetic syndrome associated with hypoglycaemia, e.g., Beckwith Wiedemann Syndrome
- Gastrointestinal disorder likely to affect feed tolerance
- Planned or likely neonatal surgery
- Confirmed sepsis (culture of pathogenic organism from blood, CSF or urine)
- Hypoxic ischaemic encephalopathy
- Family history of congenital hyperinsulinism
- Suspected inborn error of metabolism
- Triplets

Exclusions are expected to be uncommon.

Twins may be included and will be individually randomised. If more than 10% of the sample involves twins, sample size assumptions will be reviewed.

Eligibility will be based only on true BGC, either by gas analyser (portable or laboratory) or laboratory chemical analyser.

2.1.3 Interventions

Following written, informed parental consent, babies will be allocated via the online randomisation system to one of the following two interventions:

Diazoxide

The active intervention will be compounded by the hospital trial pharmacist by adding five 100 mg diazoxide capsules to 50 ml of Ora Blend (standard paediatric compounding solution), giving a concentration of 10 mg/ml. Babies will be loaded with 5 mg/kg (0.5 ml/kg) orally or by gastric tube and then commenced on a maintenance dose of 1.5 mg/kg (0.15 ml/kg) every 12 h. These doses are at the lower end of the range recommended in the NZ Formulary for Children. Although infants with congenital hyperinsulinism usually receive higher maintenance doses of 5-10 mg/kg/day, our clinical experience has shown that this is often too high for babies with transitional hypoglycaemia and may cause hyperglycaemia, whereas lower doses are similarly efficacious but avoid high BGC.[11] A bedside algorithm will be used to titrate the study drug according to BGC, commencing immediately before the third maintenance dose (see below). Once the primary outcome is reached, one further dose of study drug will be given and then the intervention will be discontinued. It may also be stopped prior to the primary outcome being reached as per the titration protocol. Weekly dose adjustment for weight will be made, if required, once the baby returns to birthweight.

Placebo

The control intervention will consist of an equal volume of Ora Blend (0.5 ml/kg load, 0.15 ml/kg maintenance), combined with a small amount of corn-starch. Our bench studies have shown that this placebo is identical in appearance to the diazoxide solution and both maintain similar physical characteristics at room temperature for at least 2 weeks. The glucose load from the corn-starch is trivial and will not affect BGC. Dosing and discontinuation will be as per diazoxide.

Supply of the intervention

Study interventions will be prepared by the hospital trial pharmacist every 2 weeks, but once formal stability test data are available, it is likely this can be extended to every 3-4 weeks.

2.1.4 Glucose and Fluid Management After Randomisation

Blood glucose target and monitoring

At all times, target BGC 2.6-5.4 mmol/L. This target range is based on normative data from the GLOW Study.[40]

Management decisions will be based only on true BGC, either by gas analyser (portable or laboratory) or laboratory chemical analyser. Because gas analysers provide plasma-equivalent glucose concentration, whole blood gas analyser and laboratory plasma measurements will be used interchangeably without adjustment.[41] Capillary, arterial or venous blood samples are acceptable.

BGC should be measured at least every 6 hours (pre-feed if on enteral bolus feeding) until the primary outcome is reached. Under special circumstances the frequency of BGC testing may be reduced before achieving the primary outcome, after discussion with the site Principal Investigator. However, to achieve the primary outcome a minimum of four pre-feed BGC in the target range of 2.6 to 5.4 mmol/L are required over ~24 h (see below).

Once the primary outcome has been achieved, BGC measurement frequency will be at clinical discretion but should be at least 12-hourly while on study drug or continuous glucose monitoring (CGM).

Fluids and feeds

Management of fluids and feeding will be as per local practice but with the aim of weaning intravenous fluids and introducing enteral feeds as soon as possible once BGC have stabilised. See below for management options if BGC goes outside the target range of 2.6-5.4 mmol/L.

Hypoglycaemia

Episodes of hypoglycaemia after randomisation (<2.6 mmol/L on blood gas or laboratory chemical analyser) will be managed according to local practice, which could include buccal dextrose gel, increasing enteral feed volume or frequency, and starting or increasing intravenous dextrose fluids. If hypoglycaemia occurs once the intervention titration algorithm has commenced (immediately before the third maintenance dose), the maintenance dose may be increased (see titration algorithm below).

Glucagon injections should only be used in emergencies where intravenous access cannot be obtained and BGC persists <1.2 mmol/L. Glucagon infusions are not permitted.

Glucocorticoids are not permitted for treatment of hypoglycaemia but may be used if deemed essential for management of other conditions, e.g., adrenal insufficiency.

Open label diazoxide may be considered in refractory cases once other management strategies have been maximised and after discussion with the attending neonatologist, Site Principal Investigator and a paediatric endocrinologist. This will require unblinding of treatment allocation, which should generally not occur before 2 weeks of age.

Elevated glucose or hyperglycaemia

Wherever possible, aim to avoid episodes of elevated blood glucose (5.5-6.9 mmol/L) or hyperglycaemia (≥7 mmol/L). If this occurs prior to commencing the intervention titration algorithm (immediately before the third maintenance dose) consider the following:

- If on intravenous fluids, wean by 50% or stop.
- If on formula or expressed breast milk supplements while establishing breastfeed, stop supplements for E or F feeds and decrease volume for A-D feeds.

Once the intervention titration algorithm has commenced (immediately prior to the third maintenance dose), continue to wean intravenous fluids or supplementary feeds as above, and withhold/decrease/stop the intervention as determined by the algorithm. If there are difficulties following the algorithm, consult with the Site Primary Investigator.

2.1.5 Titration of the Intervention

A bedside algorithm will be used to titrate the study drug according to BGC, commencing immediately before the third maintenance dose (Table 2). When commencing the bedside algorithm, refer to last BGC, which must have been performed within the last 6 hours. Based on the last BGC follow the red, green or orange zones of the algorithm.

Consult the algorithm before each maintenance dose; a BGC must have been performed within the last 6 hours to use the algorithm.

Table 2: Intervention beside algorithm: commence immediately prior to third maintenance dose (excluding loading dose) and review prior to each subsequent dose.

**BGC**	**Third maintenance dose: refer to BGC over last 6 h**	**Subsequent maintenance doses: refer to BGC over the last 12 h**
≤2.5 mmol/L	• Increase maintenance dose to 0.25 ml/kg (diazoxide 2.5 mg/kg) every 12 h and adjust fluids and feeds as clinically appropriate.<br>- If any hypoglycaemia occurs after two doses of study drug at 0.25 ml/kg, increase maintenance dose to 0.5 ml/kg (diazoxide 5.0 mg/kg) every 12 h.<br>- If any hypoglycaemia occurs after two further doses of study drug at 0.5 ml/kg, discuss with the Site Principal Investigator and a paediatric endocrinologist.	• If any hypoglycaemia has occurred over the last 12 h increase maintenance dose to 0.25 ml/kg (diazoxide 2.5 mg/kg) every 12 h and adjust fluids and feeds as clinically appropriate.<br>- If any hypoglycaemia occurs after two doses of study drug at 0.25 ml/kg, increase maintenance dose to 0.5 ml/kg (diazoxide 5.0 mg/kg) every 12 h.<br>- If any hypoglycaemia occurs after two further doses of study drug at 0.5 ml/kg, discuss with the Site Principal Investigator and a paediatric endocrinologist.
2.6-5.4 mmol/L	• Continue maintenance dose every 12 h while weaning intravenous fluids and grading up feeds.<br>• Give one more dose after the primary outcome point is reached.	• Continue maintenance dose every 12 h while weaning intravenous fluids and grading up feeds.<br>• Give one more dose after the primary outcome point is reached.
5.5-6.9 mmol/L	• If on intravenous dextrose, stop or wean fluids more rapidly OR if not on intravenous dextrose and breastfeeding, stop or wean any formula feeds<br>• Withhold intervention dose and review in 12 hours.<br>- If BGC does not return to and remain in the target range (2.6-5.4 mmol/L) over the next 12 h, discontinue the intervention.<br>- If BGC returns to the target range with no further elevated BGC over the next 12 h, recommence next maintenance at 0.1 ml/kg (diazoxide 1 mg/kg) every 12 h. If further elevated BGC occurs, discontinue the intervention.	• If any elevated BGC has occurred over the last 12 h stop intravenous dextrose and supplementary feeds if breastfeeding.<br>• If the maintenance dose is 0.1 ml/kg (diazoxide 1 mg/kg) every 12 h, discontinue the intervention.<br>• If the maintenance dose is 0.15 ml/kg (diazoxide 1.5 mg/kg) every 12 h, withhold intervention dose and review in 12 hours.<br>- If BGC does not return to and remain in the target range (2.6-5.4 mmol/L) over the next 12 h, discontinue the intervention.<br>- If BGC returns to the target range with no further elevated BGC over the next 12 h, recommence next maintenance at 0.1 ml/kg (diazoxide 1 mg/kg) every 12 h.<br>• If the maintenance dose is >0.15 ml/kg (diazoxide 1.5 mg/kg) every 12 h, withhold intervention dose and review in 12 hours.<br>- If BGC does not return to and remain in the target range (2.6-5.4 mmol/L) over the next 12 h, discontinue the intervention.<br>- If BGC returns to the target range with no further elevated BGC over the next 12 h, recommence next maintenance at 0.15 ml/kg (diazoxide 1 mg/kg) every 12 h.
≥7 mmol/L	• Discontinue the intervention.	• If any hyperglycaemia (BGC ≥7 mmol/L) has occurred over the last 12 h, discontinue the intervention.

2.1.6 Continuous Glucose Monitoring

Babies enrolled in the trial will have a subcutaneous real-time CGM sensor inserted in the lateral thigh (Medtronic Guardian 3). It will be calibrated four times in the first 24 h, then every 12 h using BGC in the target range (2.6-5.4 mmol/L). Calibration will be avoided when sensory glucose concentration (SGC) is changing rapidly (Rise or Fall Alert).[41] Using Bluetooth transmission to a bedside tablet computer and remote cloud monitoring with text alerts, research staff will use pre-defined Trend Alarms to inform the bedside nurse that a BGC measurement is indicated, i.e., glucose is trending out of range (Table 3).

Table 3: Trend Alarms

Trend Alarm	Medtronic Guardian setting	Interpretation
Low	Low Alert SGC=3.1 mmol/L AND Fall Alert ≥1 for 10 min*	BGC expected to be 2.5 mmol/L
	Low Alert SGC=3.1 AND ≤2.5 mmol/L after 20 min**	BGC falling by 0.03 mmol/L/min
High	High Alert SGC=5.6 mmol/L AND Rise Alert ≥1 for 10 min*	BGC expected to be 6.2 mmol/L
	High Alert SGC=5.6 mmol/L AND ≥6.1 mmol/L after 20 min***	BGC rising by 0.03 mmol/L/min

Medtronic Guardian provides an SGC reading ever 5 min. SGC/BGC, sensor/blood glucose concentration. *Fall/Rise Alert 1 indicates SGC is changing by 0.06 mmol/L/min; Fall/Rise Alert 2 indicates SGC is changing by 0.11 mmol/L/min; Fall/Rise Alert 3 indicates SGC is changing by 0.17 mmol/L/min. **If the SGC is ≥2.6 after 20 min, no Trend Alert is signaled. Snooze time for device Low Alert set to 30 min. ***The BGC 97^{Th} percentile for healthy breastfed babies >72 h is 6.0 mmol/L.[40] The High Alert was not signaled within 1 hour of commencement of a feed as an increase in BGC on feeding is a normal physiological response.

Actual SGC will not be reported to clinical staff, ensuring that management decisions are based solely on BGC. The CGM will remain in place for 24 h after discontinuation of the study drug or attainment of the primary outcome, whichever is longer, up to a maximum of 7 days. We have extensive experience with CGM, and have found these devices to be well tolerated in neonates (the subcutaneous filament is soft and <0.4 mm thick).[22,35,36] CGM alert and SGC data will be recorded with all BGC measurements for later agreement analysis.

2.1.7 Other Co-interventions

All other neonatal care will occur according to local practice.

2.1.8 Study Assessments

Baseline data

Demographic, obstetric and relevant family medical history will be collected at study entry. Participant ethnicity will be recorded according to Ministry of Health guidelines.[42] For participants that identify as Māori, iwi and hāpu will be recorded.

Blood tests

As part of routine care, blood will be collected at baseline and sent to the hospital laboratory for measurement of metabolic markers, including plasma insulin, beta-hydroxybutyrate, free fatty acids, creatinine and blood gas. This is standard practice for babies admitted to neonatal intensive care with hypoglycaemia.[43] All infants will have a standard metabolic screen by Guthrie card at ≥48 h as part of routine care.

Additional heparinised blood (~3 ml) will be collected before the third study maintenance dose (36 h after commencing the intervention) and plasma stored for measurement of insulin, creatinine and

diazoxide concentrations (2x 150 µL aliquots). Where possible, this will be timed to coincide with other routine blood sampling.

Cardiac ultrasound

At Middlemore Hospital, a cardiac ultrasound will be performed ≥72 hours after commencing the study intervention to assess a) ductal patency, flow and shunt; b) pulmonary arterial pressure; and c) cardiac function. Images will be stored and measured off-line according to a standardised protocol. At other sites, cardiac ultrasound will be performed as clinically indicated.

Primary and secondary outcome data

BG results and fluid and feeding charts will be reviewed regularly and recorded on a flow sheet until primary hospital discharge to determine the primary and secondary outcomes. If the primary outcome has not occurred after four weeks, it will be censored.

2.1.9 Outcomes

Primary

The primary outcome is time to resolution of hypoglycaemia, defined as achieving enteral bolus feeding and normal glucose concentrations without intravenous fluids. The primary outcome is the first time point at which all the following criteria are met concurrently (see appendix 5.6 for examples):

1) No intravenous fluids for ≥24 h (time recorded at the end of the 24-hour period).
2) Enteral bolus feeding for ≥24 h defined as a) breastfeeding without supplements; or b) breastfeeding with supplements at >2 hourly intervals, or c) if not breastfeeding, gastric tube or bottle feeds at 3-4 hourly intervals (time recorded at the end of the 24-hour period).
3) Glucose stabilisation for ≥24 h, defined as a minimum of four pre-feed BGC in the target range of 2.6 to 5.4 mmol/L (last BGC <4 h of primary outcome time point; four pre-feed BGC spanning >20 h; no BGC out of range for ≥24 h; time recorded at the end of the period).

Secondary

The following secondary outcomes will be assessed from the point of randomisation:

1) Time to establish glucose stabilisation for ≥24 h, defined as a minimum of four pre-feed BGC in the target range of 2.6 to 5.4 mmol/L (last BGC within 4 h; four pre-feed BGC spanning >20 h; no BGC out of range for ≥24 h; time recorded at the end of the period).
2) Time to establish enteral bolus feeding for ≥24 h, defined as a) breastfeeding without supplements; or b) breastfeeding with supplements at >2 hourly intervals, or c) if not breastfeeding, gastric tube or bottle feeds at 3-4 hourly intervals (time recorded at the end of the 24-hour period).
3) Time to establish full sucking feeds for ≥24 h, defined as ≥five full (code E/F) breastfeeds in 24 hours or ≥120 ml/kg/d of expressed breast milk or formula by bottle (up to discharge to home).
4) Feeding at discharge from hospital and to home .
5) Use of intravenous fluids and type.
6) Duration of intravenous fluids (up to discharge from hospital).
7) Episodes of hypoglycaemia (<2.6 mmol/L), elevated glucose concentration (5.5 to 6.9 mmol/L) and hyperglycaemia (≥7 mmol/L),[44] defined by BG measurement, including frequency, duration, timing and treatment before, during and after the episode (up to discharge from hospital).
8) Number of BG tests: during study intervention and hospital admission.
9) Duration of admission: neonatal care, postnatal ward, community birthing unit.
10) Duration of study intervention (up to discharge from hospital).

11) plasma insulin, beta-hydroxybutyrate, free fatty acids, creatinine concentrations and blood gas (on admission to the neonatal unit).
12) Guthrie metabolic screen (≥48 hours from birth).
13) Plasma insulin, creatinine and diazoxide concentrations at ≥36 hours after commencing the intervention.
14) Death (up to discharge from hospital).
15) Seizures (total; hypoglycaemic) (up to discharge from hospital).
16) Discontinuation of study intervention due to elevated BG concentration or hyperglycaemia (up to discharge from hospital).
17) Discontinuation of study intervention due to another adverse event (serious; non-serious) (up to discharge from hospital).
18) Congestive heart failure (respiratory distress as evidenced by tachypnoea, recession, or use of oxygen or positive pressure support with consistent CXR findings, including cardiomegaly, plethora, interstitial fluid or effusions) (up to discharge from hospital).
19) Commencement of low flow oxygen or positive pressure respiratory support (up to discharge from hospital).
20) Cardiac ultrasound (Middlemore Hospital) at (≥72 hours):
    a. Ductus arteriosus: closed; trivial (<1.5 mm 2D, a constricted pattern on Doppler); patent (≥1.5 mm, growing, pulsatile or bidirectional pattern on Doppler)
    b. Pulmonary hypertension: pulmonary artery pressure ≥systemic as estimated by tricuspid regurgitant jet (RV-RA gradient +5 mmHg) or ductal shunt right to the left (>20%) with characteristic pulmonary Doppler envelope (TPV/ RVET <20%)
    c. Cardiac impairment: left ventricular internal diameter diastole z-score >2 and reduced systolic function (FS% <25 or MPI >0.41)

2.1.10 Participant Timeline

The study schedule is as follows:

	**Enrolment**	**Allocation**			
**TIMEPOINT**	***$-t_1$***	**0**	***Week 1***	***Week 2-4***	***Discharge***
**ENROLMENT:**					
Eligibility screen	X				
Informed consent	X				
Baseline data	X				
Demographics and contacts	X				
Baseline metabolic bloods	X				
Allocation		X			
**INTERVENTIONS:**					
Study drug			X	±	
**ASSESSMENTS:**					
Continuous glucose monitor			X		
Primary outcome assessment			X	±	
Blood collection (≥36 hours)			X		
Echocardiogram (≥72 hours)			X		

Secondary outcome assessment			X	X	X

2.1.11 Sample Size and Feasibility

A trial of 74 babies randomised in 1:1 ratio (37 per group), will give 80% power to detect a relative hazard of 2.0 (2-tailed alpha 0.05), assuming 90% of infants in each group have a primary outcome event within the study period (PAS v.16). A hazard ratio of 2.0 indicates that the diazoxide group reaches the primary outcome at twice the rate (events per unit of time) of the control group.

Approximately 50 babies ≥35 weeks' gestation are admitted with hypoglycaemia each to the Middlemore and Auckland City Hospital neonatal units annually; thus, recruitment is feasible over 2 years at 40% recruitment rate or 18 months with the addition of a third site.

2.1.12 Recruitment

Recruitment will be face to face. It is anticipated that parental informed consent will be obtained after birth. Randomisation will only occur once a neonate is admitted to neonatal care and meets the inclusion criteria. The study will be promoted in hospital posters, flyers and the BABBLE phone application.

2.2 Assignment of Interventions

2.2.1 Allocation Sequence Generation

A computer-generated randomisation sequence with random permuted blocks of 4 and 6, stratified by centre and SGA status (<10th customised centile)[45] will be used to assign study interventions.

2.2.2 Allocation Concealment Mechanism

Participants will be assigned to study interventions using a web-based computer randomisation system (Central Coordinating Research Hub [CDRH], Liggins Institute). The randomisation system will assign a study drug bottle identified by a random number, containing either diazoxide or placebo. Only the study statistician and data manager will have access to the allocation sequence during the trial, and only the data manager and trial pharmacists will know the contents of the bottles.

2.2.3 Implementation

Participants will be enrolled and randomised by study personnel or neonatal unit staff.

2.2.4 Blinding (Masking)

Study personnel, clinical staff and parents will be blinded to the study interventions until all participants have completed the assessment period. Blinding will be achieved by using an identical medication bottle and identical inert placebo.

2.2.5 Emergency Unblinding

The attending neonatologist may request unblinding if this is deemed essential for the participant's ongoing clinical care. The Site Principal Investigator will decide to unblind after discussion with the attending neonatologist and a paediatric endocrinologist (if relevant). If unblinding occurs, the data manager will inform the attending neonatologist directly of the study intervention status; where possible, study personnel will remain blinded until the trial is completed. Unblinding should generally not be considered until at least two weeks of age.

2.3 Data Collection, Management and Analysis

2.3.1 Data Collection Methods

Data will be collected directly into eCRFs using the REDCap system. Branching logic and range checks will be used to reduce data entry errors. CGM data will be captured in a secure cloud account and subsequently uploaded to the REDCap system.

2.3.2 Retention

If a participant is withdrawn, consent will be sought to use data collected up to the point of withdrawal. Where possible, reduced participation (stopping the intervention or not performing certain assessments) will be sought rather than withdrawal. Participants who withdraw but give consent for the use of collected data will be included in the intention to treat analysis, censored at the point of withdrawal.

2.3.3 Data Management

The CDRH, Liggins Institute will provide web-based data management. All eCRFs will be manually checked for completeness and logic errors by a data monitor, after which eCRFs will be locked. If the data monitor identifies potential errors, an electronic query will be raised and referred to the site for checking.

2.3.4 Statistical Methods

Statistical analysis will be performed with JMP v14 and SAS v9.4 (SAS Institute).

Derived variables

Customised birthweight centiles will be calculated using GROW software (Perinatal Institute, United Kingdom). Population z-scores for weight, length and head circumference at birth will be calculated using UK-WHO centiles.[46]

Descriptive statistics

Categorical data will be presented as number and percent, and continuous data as mean and standard deviation or median and inter-quartile range, as appropriate. Count data will be presented as median and inter-quartile range or grouped into ordinal categories. Denominators will be given for all outcomes.

Primary analysis

Intervention groups will be compared for the primary outcome using Cox's proportional hazards regression analysis, with treatment effect expressed as hazards ratio with a 95% confidence interval (CI). The analysis will be left-censored for 24 hours and right-censored at four weeks. Secondary outcomes will be compared between groups using generalised linear models with treatment effect presented as odds ratio, count ratio, mean difference or ratio of geometric means (positively skewed data), as appropriate, with 95% CI. Regression models will be adjusted for gestation length and birthweight z-score (fixed effects), and non-independence of multiples (random effect). For significance tests, the alpha level will be set at 0.05 (two-tailed).

CGM evaluation

All BGC will be paired with CGM Trend Alert status (Low, High, Unstable, None) over the preceding 20 min. For each Trend Alert status, descriptive statistics will be presented for BGC, including mean, SD, 95% range and the proportion of values within, below and above the target range (2.6-5.4 mmol/L). Agreement analysis, expressed as kappa value with 95% CI, will be performed overall comparing the binary variables Trend Alert (yes, no) and BGC out of range (yes, no), and separately for Trend Alert low (vs. BGC below range) and high (vs. BGC above range). Kappa values of 0.41 to

0.60 will be designated as indicating moderate agreement; 0.61 to 0.80 substantial agreement; and 0.81 to 1.00 as high agreement.[47] Negative and positive predictive value for Trend Alarm will be calculated.

2.4 Monitoring

2.4.1 Data Monitoring and Safety Committee

A Data Monitoring and Safety Committee (DMSC) will monitor recruitment, completeness of data acquisition, and safety outcome measures (harms). The DMSC will advise the Steering Committee on trial continuation or protocol modification. DMSC Terms of Reference, including the content of DMSC reports, will be agreed prior to commencement of trial.

2.4.2 Interim Analysis

It is envisaged that the trial will be completed as planned with no interim efficacy analysis, but rates of adverse events will be monitored.

2.4.3 Harms

The following Serious Adverse Events (SAE) will be reported to the DMSC for immediate review:

- Death
- Seizure
- Congestive heart failure
- Discontinuation of study intervention due to another serious adverse event, as judged by the Site Principal Investigator or attending neonatologist (an adverse event is considered serious if it is immediately life-threatening, requires prolongation of existing hospitalisation or substantial escalation in care, or results in persistent or significant disability or incapacity)

The serious adverse event review process will be specified in the DMSC Terms of Reference and agreed prior to commencement of the trial.

The DMSC will undertake an interim safety review when the primary outcome is known for 25% and 60% of participants, which will include rates of SAE and the following Adverse Events (AE) by masked treatment group:

- Hyperglycaemia (≥7 mmol/L)
- Discontinuation of study intervention due to elevated BG concentration or hyperglycaemia
- Discontinuation of study intervention due to another adverse event (non-serious)
- Commencement of low flow oxygen or positive pressure respiratory support

3 ETHICS AND DISSEMINATION

3.1 Research Ethics Approval

National ethics approval will be obtained from the Health and Disability Ethics Committee (HDEC) prior to commencement of randomisation. Annual reports will be submitted to HDEC during the course of the trial.

3.2 Locality Approval

Institutional approval will be obtained at each participating District Health Board (DHB) prior to commencement of randomisation at that site.

3.3 Protocol Amendments

All amendments to the final version of this protocol will require review and approval of the Steering Committee and will be submitted to HDEC and DHB Research Offices, as appropriate. All amendments, including an approval date, will be recorded with this protocol (Appendix 5.1).

3.4 Consent

Informed written parental consent will be obtained by study personnel and clinical staff prior to enrolment in the trial.

3.5 Withdrawal

Parents will retain the right to withdraw their baby from the study at any stage without the need to provide a reason. With parental permission, data collected up to the point of withdrawal will be used in the analysis.

3.6 Ancillary Studies

Participants may be invited to participate in a parallel study of patient-centred outcomes related to neonatal hypoglycaemia, including cultural perspectives.

3.7 Confidentiality

Electronic databases will be stored on secure servers at the University of Auckland and access will be controlled by unique user ID and password, with full electronic tracking log. The screening database will record NHI, gestation length, birthweight, hypoglycaemic risk factor and eligibility criteria of participants considered for enrolment. This will enable the reporting of CONSORT data and assessment of external validity. Following randomisation, trial data will be stored in a separate database with eCRFs labelled by randomisation ID. NHI will be stored in the randomisation but not the trial database. Contact information will be stored in a separate database, independently of the trial database and will be accessible only to site coordinators and investigators. Data Access Groups will be employed so that site personnel can only see data for participants at their site. The download of data will be restricted to the data manager, study coordinator, site investigators and primary investigator, and only the data manager and primary investigator will be able to download identifiable data.

Electronic data files, e.g., CGM output, will be stored in the REDCap trial database file repository providing the same secure, protected access as above. Any hard copy CRFs will be stored in a locked cabinet until scanned into the file repository and then destroyed.

Study reports will contain only summary data and individual participant data will not be reported. Identifiable data will not be released to any third party. Research staff will be certified in best practice for clinical trials (ICH-GCP E6 and PHRP).

At the completion of the study, all electronic data will be permanently digitally archived with the CDRH, Liggins Institute, which has established processes for archiving and data sharing according to international best practice (https://wiki.auckland.ac.nz/display/ontrack/Data+Sharing).

3.8 Declaration of Interests

Investigators will declare any financial, intellectual or other potential conflicts of interest, as outlined by the ICMJE, to the Steering Committee.[48] The Steering Committee will decide on how any conflicts of interest are to be managed. This will be recorded with the Steering Committee Terms of Reference.

3.9 Access to Data

The Steering Committee will have access to the full de-identified dataset and oversee analysis, interpretation and reporting of results. Approval will be sought from the Steering Committee prior to publication of study data. Care will be taken to avoid duplication in reporting of results.

3.10 Dissemination Policy

Results of the study will be presented at relevant conferences and hospital meetings and published in a peer-reviewed scientific journal.

3.11 Authorship policy

The Council of Science Editors standards for authorship will be applied (www.councilscienceeditors.org). The Steering Committee will be responsible for planning manuscripts and determining authorship. Investigators and study personnel who do meet the criteria for authorship will be acknowledged as non-author contributors.

3.12 Data Sharing Policy

For each main publication, the corresponding data set will be electronically archived with the CDRH. Anonymised data may be shared with external researchers upon request, according to the Data Sharing Protocol of the CDRH (https://wiki.auckland.ac.nz/display/ontrack/Data+Sharing).

3.13 Māori Responsiveness

This protocol was developed in consultation with the Liggins Institute Māori Advisory Group of (2019.08.14) and Te Teira Rawiri, Principal Kaumātua, Counties Manukau Health (2019.10.16). Our investigator team includes a Māori paediatric researcher, Jenny Rogers (Ngāi Tahu), who will assist with Māori engagement, ensure that study processes are culturally appropriate and provide a Māori research perspective in analysis and dissemination of data.

4 STUDY MANAGEMENT

4.1 Steering Committee

The Steering Committee will take overall responsibility for all aspects of the study, meeting on a quarterly basis. Matters arising between meetings may be dealt with by email. The Principal Investigator will be responsible for maintaining a record of correspondence and minutes of meetings.

4.2 Management Committee

A Study Coordinator will be appointed to oversee the day-to-day running of the study. They will be supported by a Management Committee which will meet regularly.

4.3 Site Principal Investigator

A Principal / Lead Investigator will be appointed at each site, who will have overall responsibility for satisfying local governance requirements, recruitment, assessments, data collection and integrity. They will be supported by the Trial Coordinator and Management Committee.

4.4 Finance and Insurance

Funding has been provided from the University of Auckland and Health Research Council of New Zealand.

This is a non-commercial study; participants in New Zealand will be covered by provisions of ACC.

5 APPENDICES

5.1 Protocol Amendments

Version, Date	Amendment(s)	Date accepted by Steering Group	Date ethics notified (or NA)
1.1, 2019.11.26	Addition of congestive heart failure and commencement of low flow oxygen or positive pressure respiratory support as secondary outcomes and serious adverse and adverse events, respectively.		
1.2, 2019.12.22	Assessment time point updated for secondary outcomes.		
1.3, 2020.2.5	Added “phase 2” to design; labelled as study number I; appendix 5.4 updated.		
1.4, 2020.2.15	Definition of glucose stability corrected.		
1.5, 2020.2.29	Updated with feedback from the ON TRACK Trials Workshop, including minor amendments to eligibility and definition of the primary outcome.		
1.6, 2020.4.23	Michael Myer added as investigator.<br>CGM methods updated.		
1.7, 2020.5.1	Error in titration algorithm corrected.		
1.8, 2020.5.13	Target range upper limit increased to 5.4 mmol/L based on new normative data. CGM methods updated.		
1.9, 2020.9.4	Study blood volume increased to 3 ml to allow 2x 150 ul aliquots to be achieved.		
2.0, 2020.10.19	Clarification of wording of titration algorithm and co-interventions.	2020.11.30	submitted
2.1, 2020.12.10	Reference for definition of hyperglycaemia added.<br>Definitions for secondary outcomes 1-3 added.<br>Title corrected.		
2.2, 2021.03.03	Snooze for CGM alarm changed to 30 min.		
2.4, 2021.10.10	Eamon Walsh added as an investigator.		
2.5, 2021.11.11	Trend Alert changed to Trend Alarm to distinguish this factory set alerts.<br>Primary outcome reworded as resolution of hypoglycaemia (definition unchanged).<br>Trial title amended due to change in label of primary outcome.		
2.6, 2022.6.27	Updated affiliations and professional titles		

5.2 Participant Documents

The following participant documents are to accompany this protocol:

Title	Version	Date
Participant Information Sheet and Consent	1.6	2021.11.15

5.3 Ethical and Locality Approval

The following letters of approval are to accompany this protocol:

Title	Reference	Date
Medsafe: SCOTT approval not required	Email on file	2019.08.1
Liggins Institute Māori Advisory Group: consultation	Letter on file	2019.08.14
Liggins locality approval	Letter on file	2019.11.8
Counties Manukau Health locality approval	Letter on file	2020.3.11
Auckland District Health Board locality approval (8745)	Letter on file	2020.2.28
HDEC approval 19CEN189 (corrected letter)	Letter on file	2020.3.9

5.4 Study Committees

The following Terms of Reference are to accompany this protocol:

Title	Version	Date
Data and Safety Monitoring Committee Terms of Reference	1.3	2020.5.24

5.5 Standard Operating Procedures

The following Standard Operating Procedure documents are to accompany this protocol:

Title	Version	Date
How to randomise	8.1 (MMH)<br>8.1 (ACH)	2021.7.21<br>2021.02.02
Bedside guide	1.1	2021.8.12
Blood collection	1.0	2021.01.31
CGM	1.3	2021.07.31
Medication supply	1.0	2021.01.31

5.6 Funding

This study was funded through the following sources:

Funder	Funding type	Named Investigators	Amount
University of Auckland	Early Career Research Award	Chris McKinlay	$24,719
Health Research Council	Feasibility Project (20/651)	Chris McKinlay, Jane Alsweiler, Jane Harding, Wayne Cutfield, Jenny Rogers, Greg Gamble, Geoff Chase	$249,641

6 REFERENCES

1. Chan AW, Tetzlaff JM, Altman DG, Laupacis A, Gotzsche PC, Krleza-Jeric K, et al. SPIRIT 2013 statement: defining standard protocol items for clinical trials. Ann Intern Med. 2013;158(3):200-7.
2. Chan AW, Tetzlaff JM, Gotzsche PC, Altman DG, Mann H, Berlin JA, et al. SPIRIT 2013 explanation and elaboration: guidance for protocols of clinical trials. BMJ. 2013;346:e7586.
3. Harding JE, Harris DL, Hegarty JE, Alsweiler JM, McKinlay CJD. An emerging evidence base for the management of neonatal hypoglycaemia. Early Hum Dev. 2017;104:51-6.
4. Sundercombe SL, Raynes-Greenow CH, Turner RM, Jeffery HE. Do neonatal hypoglycaemia guidelines in Australia and New Zealand facilitate breast feeding? Midwifery. 2014.
5. Blomquist HK, Jonsbo F, Serenius F, Persson LA. Supplementary feeding in the maternity ward shortens the duration of breast feeding. Acta Paediatr. 1994;83(11):1122-6.
6. Glasgow MJ, Harding JE, Edlin R. Cost-analysis of treating neonatal hypoglycemia with dextrose gel. J Pediatr. 2018;doi: 10.1016/j.jpeds.2018.02.036.
7. Dassios T, Greenough A, Leontiadi S, Hickey A, Kametas NA. Admissions for hypoglycaemia after 35 weeks of gestation: perinatal predictors of cost of stay. J Matern Fetal Neonatal Med. 2019;32(3):448-54.
8. Harris DL, Weston PJ, Harding JE. Lactate, rather than ketones, may provide alternative cerebral fuel in hypoglycaemic newborns. Arch Dis Child Fetal Neonatal Ed. 2014;100(2):F161-4.
9. Riviere D, McKinlay CJD, Bloomfield FH. Adaptation for life after birth: a review of neonatal physiology. Anaesthesia Intensive Care Medicine. 2016;18(2):59-67.
10. Shah R, Harding J, Brown J, McKinlay C. Neonatal glycaemia and neurodevelopmental outcomes: a systematic review and meta-analysis. Neonatology. 2019;115(2):116-26.
11. McKinlay CJ, Alsweiler J, Anstice N, Burakevych N, Chakraborty A, Chase JG, et al. Association of neonatal glycemia with neurodevelopmental outcomes at 4.5 years. JAMA Pediatr. 2017;171(10):1-12.
12. McKinlay CJD, Alsweiler JA, Ansell JM, Anstice NS, Chase JG, Gamble GD, et al. Neonatal glycemia and neurodevelopmental outcomes at two years. N Engl J Med. 2015;373:1507-18.
13. Kaiser JR, Bai S, Gibson N, Holland G, Lin TM, Swearingen CJ, et al. Association between transient newborn hypoglycemia and fourth-grade achievement test proficiency: a population-based study. JAMA Pediatr. 2015;169(10):913-21.
14. Kerstjens JM, Bocca-Tjeertes IF, de Winter AF, Reijneveld SA, Bos AF. Neonatal morbidities and developmental delay in moderately preterm-born children. Pediatrics. 2012;130(2):e265-72.
15. Harris DL, Weston PJ, Signal M, Chase JG, Harding JE. Dextrose gel for neonatal hypoglycaemia (the Sugar Babies Study): a randomised, double-blind, placebo-controlled trial. Lancet. 2013;382(9910):2077-83.
16. Burakevych N, McKinlay CJD, Harris DL, Alsweiler JM, Harding JE. Factors influencing glycaemic stability after neonatal hypoglycaemia and relationship to neurodevelopmental outcome. Scientific Reports. 2019;9:8132.
17. McGowan JE, Chen L, Gao D, Trush M, Wei C. Increased mitochondrial reactive oxygen species production in newborn brain during hypoglycemia. Neurosci Lett. 2006;399(1-2):111-4.
18. Cowett RM, Rapoza RE, Gelardi NL. Insulin counterregulatory hormones are ineffective in neonatal hyperinsulinemic hypoglycemia. Metabolism. 1999;48(5):568-74.
19. Senniappan S, Hussain K. An evaluation of growth hormone and IGF-1 responses in neonates with hyperinsulinaemic hypoglycaemia. Intl J Endocrinol 2013;2013.

20. Pryds O, Christensen NJ, Friis-Hansen B. Increased cerebral blood flow and plasma epinephrine in hypoglycemic, preterm neonates. Pediatrics. 1990;85(2):172-6.

21. Mujsce DJ, Christensen MA, Vannucci RC. Regional cerebral blood flow and glucose utilization during hypoglycemia in newborn dogs. Am J Physiol. 1989;256(6 Pt 2):H1659-66.

22. Hay WW, Jr., Sparks JW. Placental, fetal, and neonatal carbohydrate metabolism. Clin Obstet Gynecol. 1985;28(3):473-85.

23. Denne SC, Kalhan SC. Glucose carbon recycling and oxidation in human newborns. Am J Physiol. 1986;251(1 Pt 1):E71-7.

24. Vannucci RC, Vannucci SJ. Glucose metabolism in the developing brain. Semin Perinatol. 2000;24(2):107-15.

25. Lippe G, Galzigna L, Francesconi M, Zorzi C, Deana R. Age-dependent excretion of 3-hydroxy-3-methylglutaric acid (HMG) and ketone bodies in the urine of full-term and pre-term newborns. Clin Chim Acta. 1982;126(3):291-5.

26. Avagliano L, Mascherpa M, Massa V, Doi P, Bulfamante GP. Fetal pancreatic Langerhans islets size in pregnancies with metabolic disorders. J Matern Fetal Neonatal Med. 2018:1-6.

27. Ford SP, Zhang L, Zhu M, Miller MM, Smith DT, Hess BW, et al. Maternal obesity accelerates fetal pancreatic beta-cell but not alpha-cell development in sheep: prenatal consequences. Am J Physiol Regul Integr Comp Physiol. 2009;297(3):R835-43.

28. Limesand SW, Rozance PJ. Fetal adaptations in insulin secretion result from high catecholamines during placental insufficiency. J Physiol. 2017;595(15):5103-13.

29. Boehmer BH, Limesand SW, Rozance PJ. The impact of IUGR on pancreatic islet development and beta-cell function. J Endocrinol. 2017;235(2):R63-r76.

30. Suh SW, Gum ET, Hamby AM, Chan PH, Swanson RA. Hypoglycemic neuronal death is triggered by glucose reperfusion and activation of neuronal NADPH oxidase. J Clin Invest. 2007;117(4):910-8.

31. Ennis K, Dotterman H, Stein A, Rao R. Hyperglycemia accentuates and ketonemia attenuates hypoglycemia-induced neuronal injury in the developing rat brain. Pediatr Res. 2015;77(1-1):84-90.

32. Hirose H, Maruyama H, Ito K, Kido K, Koyama K, Saruta T. Effects of diazoxide on alpha- and beta-cell function in isolated perfused rat pancreas. Diabetes Res Clin Pract. 1994;25(2):77-82.

33. Shieh CC, Coghlan M, Sullivan JP, Gopalakrishnan M. Potassium channels: molecular defects, diseases, and therapeutic opportunities. Pharmacol Rev. 2000;52(4):557-94.

34. Grill V, Radtke M, Qvigstad E, Kollind M, Bjorklund A. Beneficial effects of K-ATP channel openers in diabetes: an update on mechanisms and clinical experiences. Diabetes Obes Metab. 2009;11 Suppl 4:143-8.

35. Hussain K, Aynsley-Green A, Stanley CA. Medications used in the treatment of hypoglycemia due to congenital hyperinsulinism of infancy (HI). Pediatr Endocrinol Rev. 2004;2 Suppl 1:163-7.

36. Balachandran B, Mukhopadhyay K, Sachdeva N, Walia R, Attri SV. Randomised controlled trial of diazoxide for small for gestational age neonates with hyperinsulinaemic hypoglycaemia provided early hypoglycaemic control without adverse effects. Acta Paediatr. 2018;107(6):990-5.

37. Demirel F, Unal S, Cetin, II, Esen I, Arasli A. Pulmonary hypertension and reopening of the ductus arteriosus in an infant treated with diazoxide. J Pediatr Endocrinol Metab. 2011;24(7-8):603-5.

38. Timlin MR, Black AB, Delaney HM, Matos RI, Percival CS. Development of Pulmonary Hypertension During Treatment with Diazoxide: A Case Series and Literature Review. Pediatr Cardiol. 2017;38(6):1247-50.

39. Ortqvist E, Bjork E, Wallensteen M, Ludvigsson J, Aman J, Johansson C, et al. Temporary preservation of beta-cell function by diazoxide treatment in childhood type 1 diabetes. Diabetes Care. 2004;27(9):2191-7.

40. Harris DL, Weston PJ, Gamble GD, Harding JE. Glucose profiles in healthy term infants in the first 5 days: the Glucose in Well Babies (GLOW) Study. J Pediatr. 2020;DOI: 10.1016/j.jpeds.2020.02.079.

41. McKinlay CJD, Chase JG, Dickson J, Harris DL, Alsweiler JM, Harding JE. Continuous glucose monitoring in neonates: a review. Matern Health Neonatol Perinatol. 2017;3:18.

42. Ministry of Health. Ethnicity data protocols for the health and disability sector. Wellington: Ministry of Health, New Zealand; 2004.

43. Thornton PS, Stanley CA, De Leon DD, Harris D, Haymond MW, Hussain K, et al. Recommendations from the Pediatric Endocrine Society for evaluation and management of persistent hypoglycemia in neonates, infants, and children. J Pediatr. 2015;167(2):238-45.

44. Rozance PJ, Hay WW. Neonatal hyperglycemia. NeoReviews. 2010;11(11):e632-e9.

45. Gardosi J, Figueras F, Clausson B, Francis A. The customised growth potential: an international research tool to study the epidemiology of fetal growth. Paediatr Perinat Epidemiol. 2011;25(1):2-10.

46. Cole TJ, Williams AF, Wright CM, Group RGCE. Revised birth centiles for weight, length and head circumference in the UK-WHO growth charts. Ann Hum Biol. 2011;38(1):7-11.

47. Landis JR, Koch GG. The measurement of observer agreement for categorical data. Biometrics. 1977;33(1):159-74.

48. Drazen JM, de Leeuw PW, Laine C, Mulrow C, Deangelis CD, Frizelle FA, et al. Towards more uniform conflict disclosures: the updated ICMJE conflict of interest reporting form. BMJ. 2010;340:c3239.

NEO GLU C O
Neonatal Glucose Care Optimisation Study

Oral diazoxide versus placebo for severe or recurrent neonatal hypoglycaemia

STATISTICAL ANALYSIS PLAN

Version 1.1

Date: 2022.11.4

Approved on behalf of the NeoGluCO Steering Committee by:

Role	Signature	Date
Chris McKinlay<br>NeoGluCO Coordinating Investigator		2022.11.4
Jane Alsweiler<br>NeoGluCO Site Principal Investigator		2022.11.7

Contents

1 Introduction 3
1.1 Aim 3
1.2 Objective and hypothesis 3
1.3 Study Synopsis 3
1.4 Roles and responsibilities 4
2 Study outcomes 5
2.1 Primary outcome 5
2.2 Secondary outcomes 5
3 Statistical analysis 6
3.1 Sample size and power 6
3.1 Data quality 6
3.2 Derived variables 7
3.3 Data availability 7
3.4 Baseline characteristics 7
3.5 Descriptive statistics 7
3.6 Primary analysis 7
3.7 Secondary analysis 8
3.8 Missing data 8
3.9 Statistical software 8
4 References .... **Error! Bookmark not defined.**
5 Appendix: Shell tables and figures 9

1 Introduction

This statistical analysis plan (SAP) outlines the analysis and reporting of the Neonatal Glucose Care Optimisation (NeoGluCO) Study. It was agreed to by all members of the NeoGluCO Steering Committee prior to data lock and analysis of the results.

This SAP has been prepared with reference to the following key documents:

- NeoGluCO Protocol, v2.6, 2022.6.27
- Laing D, Walsh E, Alsweiler JM, Hanning SM, Meyer MP, Ardern J, Cutfield WS, Rogers J, Gamble GD, Chase JG, Harding JE, McKinlay CJD. Oral diazoxide versus placebo for severe or recurrent neonatal hypoglycaemia: Neonatal Glucose Care Optimisation (NeoGluCO) Study; a randomised controlled trial. BMJ Open. 2022; DOI:10.1136/ bmjopen-2021-059452.
- Gamble C, Krishan A, Stocken D, et al. Guidelines for the content of statistical analysis plans in clinical trials. JAMA. 2017;318(23):2337–2343. doi:10.1001/jama.2017.18556.
- McLeod C, Norman R, Litton E, Saville BR, Webb S, Snelling TL. Choosing primary endpoints for clinical trials of health care interventions. Contemp Clin Trials Commun. 2019;16:100486.

1.1 Aim

The aim of the NeoGluCO Study is to investigate if early use of oral diazoxide in late preterm and term neonates with severe or recurrent neonatal hypoglycaemia reduces time to resolution of hypoglycaemia defined as achieving enteral bolus feeding and normal blood glucose concentrations without intravenous fluids. If effective, such a treatment could have major benefits for neonates with severe or recurrent hypoglycaemia, including reduced length of admission and separation of mother and baby, reduced use of formula and facilitation of the earlier establishment of breastfeeding, reduced number of heel pricks for blood glucose concentration (BGC) testing, and better long-term neurodevelopmental outcomes.

1.2 Objective and hypothesis

Primary objective: To determine if early use of diazoxide in late preterm and term neonates with severe or recurrent neonatal hypoglycaemia reduces time to resolution of hypoglycaemia, defined as achieving enteral bolus feeding and normal BGC without intravenous fluids (see below).

Primary hypothesis: Early diazoxide therapy will improve glycaemic stability, allowing earlier weaning of intravenous fluids and establishment of enteral feeds.

1.3 Study Synopsis

Phase 2, Double-blinded, randomised controlled, two-arm, parallel trial of diazoxide versus placebo in neonates born at ≥35 weeks' gestation admitted to neonatal care with severe or recurrent hypoglycaemia in the first week (Table 1). Severe hypoglycaemia was defined as any BGC <1.2 mmol/L or BGC 1.2 to <2.0 mmol/L despite two doses of dextrose gel and feeding in a single episode; recurrent hypoglycaemia was defined as ≥3 episodes (one or more consecutive BGC) of hypoglycaemia <2.6 mmol/L in 48 hours. Babies were randomised to either oral diazoxide 5 mg/kg load, then 1.5 mg/kg 12 hourly or an equivalent volume of placebo. Management of feeds, fluid and hypoglycaemic episodes was according to local practice. Once glycaemic stability was achieved, the study drug was weaned by protocol. If hyperglycaemia occurred (≥7.0 mmol/L), the study drug was discontinued. The primary outcome is time

to resolution of hypoglycaemia, defined as achieving enteral bolus feeding and normal glucose concentrations without intravenous fluids (see below).

Table 1: PICOT Summary

**Participants**	Neonates ≥35 weeks admitted to the neonatal unit with severe or recurrent hypoglycaemia in the first week.
**Intervention**	Oral diazoxide 5 mg/kg loading dose, then 1.5 mg/kg 12 hourly maintenance dose, weaning by protocol.
**Control**	An equivalent volume of identical placebo.
**Primary outcome**	Time to resolution of hypoglycaemia after randomisation, defined as achieving enteral bolus feeding and normal glucose concentrations without intravenous fluids.
**Planned sample size**	74 babies randomised in 1:1 ratio, giving 80% power to detect a relative hazard of 2.0 (2-tailed alpha 0.05).
**Timing of assessment**	Assessment for the primary outcome up to 4 weeks, after which censoring occurs.

1.4 Roles and responsibilities

The NeoGluCO Steering Committee has responsibility for conduct of NeoGluCO Study and accordingly is responsible for planning the analyses of the primary trial report and the decision to publish. The NeoGluCO Steering Committee members are as follow:

- Christopher JD McKinlay, Paediatrics: Child and Youth Health, University of Auckland, New Zealand (Coordinating Investigator)
- Jane M Alsweiler, Paediatrics: Child and Youth Health, University of Auckland, New Zealand (Site Principal Investigator)
- Don Laing, Liggins Institute, University of Auckland, New Zealand
- Eamon Walsh, Liggins Institute, University of Auckland, New Zealand
- Jane E Harding, Liggins Institute, University of Auckland
- Greg D Gamble, Liggins Institute, University of Auckland, New Zealand
- Wayne S Cutfield, Liggins Institute, University of Auckland, New Zealand
- Jenny Rogers, Liggins Institute, University of Auckland, New Zealand
- J Geoffrey Chase, College of Engineering, University of Canterbury
- Sara M Hanning, School of Pharmacy, University of Auckland
- Julena Ardern, Kidz First Neonatal Care, Te Whatu Ora Counties Manukau
- Michael P Meyer, Kidz First Neonatal Care, Te Whatu Ora Counties Manukau

All Steering Group members will have the opportunity to be authors. The first draft of the manuscript will be prepared by a writing group (CJDM, JAM, DL, EW, GDG). All authors will contribute to drafting and finalising the manuscript.

The following members of the research team will be acknowledged as non-author contributors:

- Data Monitoring and Safety Committee: Prof. Stuart Dalziel (Chair), Prof. Rinki Murphy, A/Prof. Nicola Austin
- Study coordinator: Lisa Mravicich
- Research nurse: Sabine Huth
- Pharmacists: Lisa Chen, Michelle Ure
- ON TRACK Network research advisors: Lex Doyle, Rebecca Simmons

2 Study outcomes

2.1 Primary outcome

The primary outcome is time to resolution of hypoglycaemia, defined as the first time point after randomisation at which all of the following criteria are met concurrently:

- No intravenous fluids for ≥24 hours (time recorded at the end of the 24- hour period).
- Enteral bolus feeding for ≥24 hours, defined as (a) breast feeding without supplements; or (b) breast feeding with supplements at >2 hourly intervals, or (c) if not breast feeding, gastric tube or bottle feeds at 3–4 hourly intervals (time recorded at the end of the 24- hour period).
- Normoglycaemia for ≥24 hours, defined as a minimum of four pre- feed BGC in the target range of 2.6–5.4 mmol/L spanning >20 hours (last BGC measured within 4 hours of primary outcome time point) and no BGC out of range for ≥24 hours (time recorded at the end of the period).

2.2 Secondary outcomes

The following secondary outcomes will be reported from randomisation:

- Time to achieve normoglycaemia (as per primary outcome).
- Time to establish enteral bolus feeding (as per primary outcome).
- Time to establish full sucking feeds defined as ≥5 full breast feeds (≥10 min) in 24 hours or ≥120 mL/kg/ day of expressed breast milk or formula by bottle (up to discharge to home).
- Feeding at discharge to home: exclusively breastfed from birth; full breastmilk feeds in the past 48 hours; mixed breastmilk and formula feeds in the past 48 hours.
- Use of intravenous fluids before discharge from hospital, including type of fluids.
- Duration of intravenous fluids before discharge from hospital.
- Episodes of hypoglycaemia (BGC<2.6 mmol/L), elevated glucose concentration (BGC 5.5–6.9 mmol/L) and hyperglycaemia (BGC ≥7 mmol/L) before discharge from hospital, including frequency, duration, timing and treatment of episodes.
- Number of blood glucose tests during the study intervention and hospital admission.
- Duration of admission to discharge from neonatal care and to home.
- Duration of study intervention up to discharge from hospital.

- Abnormal Guthrie metabolic screen (≥48 hours from birth).
- Plasma insulin, creatinine and diazoxide concentrations at ≥36 hours after commencing the intervention.
- Death before hospital discharge.
- Seizures before hospital discharge (total and presumed hypoglycaemic).
- Discontinuation of study drug before hospital discharge due to elevated BGC or hyperglycaemia.
- Discontinuation of study drug before discharge from hospital due to adverse event (serious; non-serious).
- Congestive heart failure (respiratory distress as evidenced by tachypnoea, recession or use of oxygen or positive pressure support with consistent chest X- ray findings, including cardiomegaly, plethora, interstitial fluid or effusions) (up to discharge from hospital).
- Commencement of low flow oxygen or positive pressure respiratory support up to discharge from hospital.
- Cardiac ultrasound (Middlemore Hospital) at (≥72 hours).
    - Ductus arteriosus: closed; trivial (<1.5 mm and a constricted pattern on Doppler); patent (≥1.5 mm, growing, pulsatile or bidirectional pattern on Doppler).
    - Pulmonary hypertension: pulmonary artery pressure ≥systemic as estimated by tricuspid regurgitant jet (right ventricular- atrial gradient +5 mm Hg) or ductal shunt right to left (>20%) with characteristic pulmonary Doppler envelope (time to peak velocity/right ventricular ejection time <20%).
    - Cardiac impairment: left ventricular internal diameter diastolic z- score >2 and reduced systolic function (fractional shortening <25% or myocardial performance index >0.41).

3 Statistical analysis

3.1 Sample size and power

We estimated that a trial of 74 babies randomised in a 1:1 ratio (37 per group) would give 80% power to detect a relative hazard of 2.0 (two-tailed alpha 0.05), assuming 90% of infants in each group have a primary outcome event within the study period (PASS Software V.16). A hazard ratio of 2.0 indicates that the diazoxide group reaches the primary outcome at twice the rate (events per unit of time) of the control group. An adaptive sample size approach was adopted where the number of randomised participants was increased by the number of participants who withdraw or who were lost to follow-up.

3.2 Data quality

Study data were collected directly into electronic case record forms (eCRF) in the REDCap system. Range and logic checks were used to reduce data entry errors. CGM data were captured in a secure cloud account and subsequently uploaded to the REDCap system. All eCRF were monitored for completeness and logic errors, after which eCRFs were locked. If potential errors were identified, an electronic query was raised and referred to the site for checking. All data queries were resolved by discussion with the research personnel and/or the Coordinating Investigator, following which the eCRF

was locked.

Before analysis, continuous data will be plotted, and outliers will be further checked for potential errors. Any corrections will be made in REDCap before export of data for analysis.

3.3 Derived variables

The following baseline variables will be calculated:

- Customised birthweight centiles using GROW software (Perinatal Institute, UK).

3.4 Data availability

A CONSORT diagram (Figure 1) will summarise the flow of infants from trial entry to assessment of the primary outcome. It will report the number of infants who were:

- Screened
- Not eligible
- Eligible but not recruited, with reasons
- Randomised into the trial
- Randomised in error
- Randomised to the diazoxide and placebo gel groups
- Did not receive intervention by treatment group
- Stopped intervention outside protocol by treatment group
- Died, withdrew, lost to follow-up by treatment group
- Assessed for the primary outcome by treatment group

3.5 Baseline characteristics

Descriptive statistics for maternal and infant baseline (pre-randomisation) characteristics will be presented for the diazoxide and placebo groups, including mean (SD) or median (IQR) for continuous outcomes, as appropriate, and number (%) for categorical variables (Table 2). The potential impact of any imbalance in baseline factors will be judged on clinical grounds.

3.6 Descriptive statistics

Categorical data will be presented as number and percent, and continuous data as mean and standard deviation or median and inter-quartile range, as appropriate. Count data will be presented as median and inter-quartile range or grouped into ordinal categories. Denominators will be given for all outcomes.

3.7 Primary analysis

All infants who meet eligibility will be included in the primary analysis (modified intention-to-treat analysis). Intervention groups will be compared for the primary outcome using Cox's proportional hazards regression analysis, with treatment effect expressed as adjusted hazards ratio with a 95%

confidence interval (CI). The analysis will be left truncated by 24 hours and right censored at four weeks. Proportionality assumptions will be assessed by inspecting Kaplan-Meier curves and Martingale residuals. The model will be adjusted for stratification variables (centre and customised birth weight centile) and gestation length. If the proportion of multiples is >10%, the model may be additionally adjusted for the non-independence of multiples.

Secondary outcomes will be compared between groups using generalised linear models (normal, binomial or Poisson) with treatment effect presented as adjusted mean difference, ratio of geometric means (positively skewed data), odds ratio or count ratio, as appropriate, with 95% CI. Regression models will be adjusted as above. If models fail to converge, the analysis algorithm will be optimised and the maximum number of iterations increased to get convergence with minimum Akaike information criteria. If this is unsuccessful, adjustment variables may be collapsed or excluded, if necessary, for model convergence. No adjustment will be made for multiple comparisons but results for secondary outcomes will be interpreted cautiously.

3.8 Secondary analysis

Secondary exploratory analysis of the primary outcome may include per-protocol analysis (intervention provided without major protocol deviation) and adjustment for any baseline prognostic variables with imbalance between groups.

3.9 Missing data

Missing outcome data will not be imputed.

3.10 Statistical software

All analyses will be performed using SAS version 9.4 (SAS Institute Inc, Cary NC, USA) unless otherwise specified.

4 Appendix: Shell tables and figures

Figure 1: Participant flow in the NeoGluCO Study

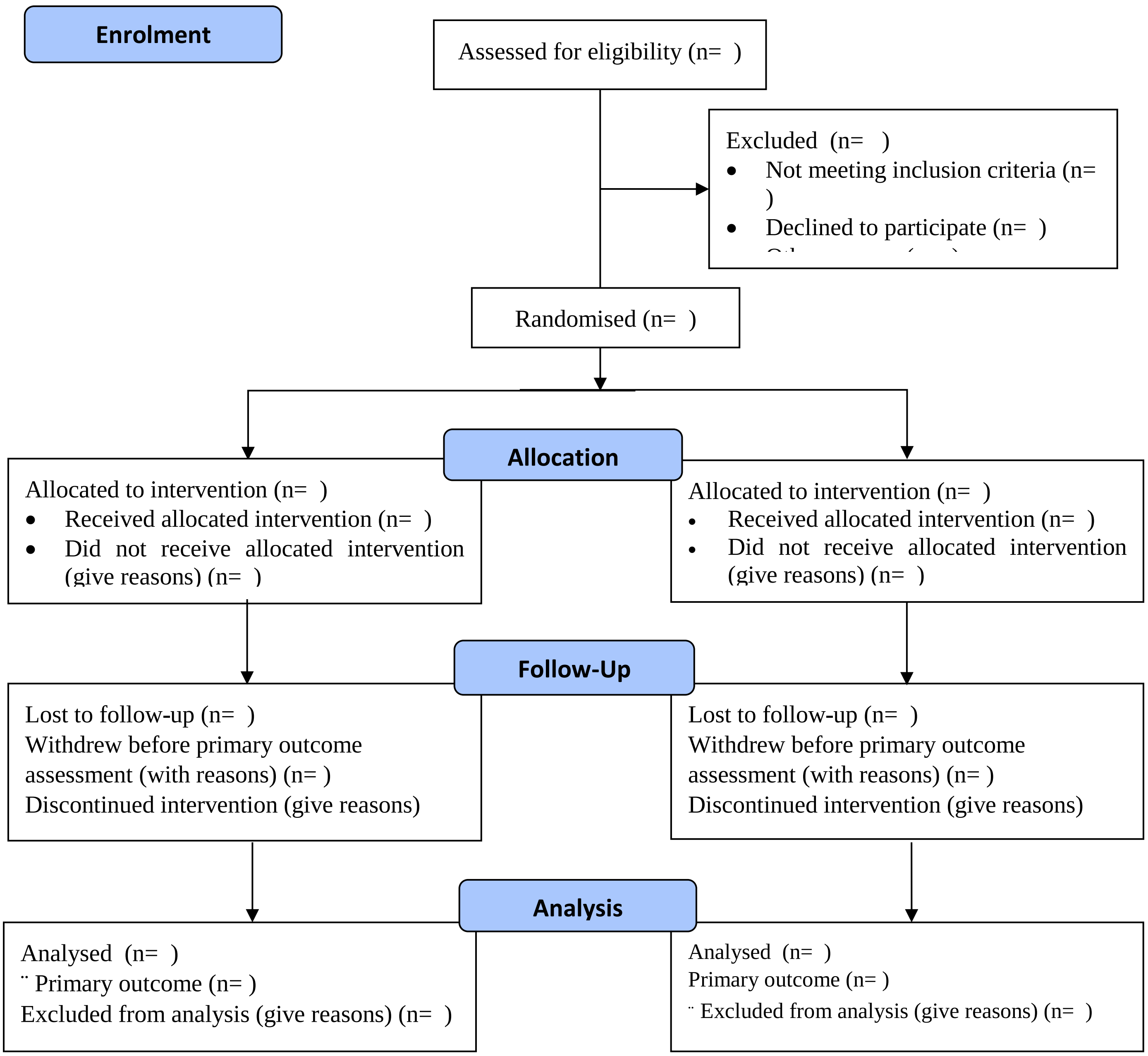

Table 1. Baseline characteristics of infants, and their mothers, in the NeoGluCO Study

Characteristic	Diazoxide	Placebo
Maternal<br>Age—years	N=	N=
Parity		
Height—m		
BMI at booking—kg/m^2<br>Obesity (>30 kg/m^2)		
Gestational weight gain—kg		
HbA1c < 20 weeks—mmol/mol		
Diabetes<br>Pregestational<br>Gestational		
Pre-eclampsia		
Hypertensive disorders of pregnancy		
Infants<br>Female	N=	N=
Multiple pregnancy		
Fetal growth restriction (obstetric diagnosis)		
Caesarean birth<br>Emergency caesarean		
Gestation—weeks<br>Preterm (<37 weeks' gestation)		
Birthweight—g		
Birthweight—customised centile		
Small for gestational age (customised birthweight centile <10)		
Large for gestational age (customised birthweight centile >90)		
Apgar score <7 at 5 minutes		
Prioritised ethnicity<br>Māori<br>Pacific<br>Indian<br>Other<br>NZ European		
Eligibility criteria<br>≥3 episodes of hypoglycaemia <2.6 mmol/L in 48 hours<br>Blood glucose of 1.2 to <2.0 mmol/L persisting after 2 doses of dextrose gel and feeding<br>≥1 episode of hypoglycaemia <1.2 mmol/L		
Admission metabolites<br>Age at testing—hours		
Insulin—mU/L		
Insulin:glucose ratio—U/mol		
β-hydroxybutyrate—mmo/L<br><0.1 mmol/L		

Free fatty acids—mE/L		
Lactate—mmol/L		
Plasma creatinine—mmol/L		
Age at randomisation—hours		

Figure 2: Primary outcome

Table 2: Secondary outcomes

	Diazoxide	N	Placebo	N	Adjusted MD, RGM, OR or CR (95% CI)
Time to achieve normoglycaemia					
Time to establish enteral bolus feeding—days					
Time to establish full sucking feeds—days					
Feeding at discharge to home<br>Exclusively breastfed from birth<br>Full breastmilk feeds in the past 48 hours<br>Mixed breastmilk and formula feeds in the past 48 hours					
Use of intravenous fluids before discharge from hospital<br>Dextrose concentration ≥15%					
Duration of intravenous fluids before discharge from hospital—days					
Hypoglycaemia (BGC<2.6 mmol/L) before discharge from hospital<br>Number of episodes<br>Number of episodes >48 hours after randomisation<br>Total duration of episodes—hours<br>Study drug increased for hypoglycaemia					
Elevated glucose concentration (BGC 5.5–6.9 mmol/L) before discharge from hospital<br>Number of episodes<br>Number of episodes >48 hours after randomisation<br>Total duration of episodes—hours<br>Study drug stopped for elevated glucose					
Hyperglycaemia (BGC ≥7 mmol/L) before discharge from hospital<br>Number of episodes<br>Number of episodes >48 hours after randomisation					

Total duration of episodes—hours<br>Study drug stopped for hyperglycaemia					
Number of blood glucose tests during the study intervention					
Number of blood glucose tests during hospital admission					
Duration of admission to neonatal unit discharge—days					
Duration of admission to discharge to home—days					
Abnormal Guthrie metabolic screen					
Plasma insulin concentration 36 hours after intervention—mU/L					
Plasma creatinine concentration 36 hours after intervention—µmol/L					
Plasma diazoxide trough concentration					
Death before hospital discharge					
Seizures before hospital discharge<br>Due to hypoglycaemia					
Congestive heart failure					
Commencement of low flow oxygen or positive pressure respiratory support					
Discontinuation of study drug before hospital discharge due to non-serious adverse event					
Discontinuation of study drug before hospital discharge due to serious adverse event					
Duration of study intervention—days					
Cardiac ultrasound<br>Patent ductus arteriosus (non-trivial)					
Pulmonary hypertension					
Cardiac impairment					

Data are number (precent), mean (standard deviation) or median (interquartile range). CI, confidence interval; CR< count ratio, MD, mean difference; OR, odds ratio. Full sucking feeds defined as ≥5 full breast feeds (≥10 min) in 24 hours or ≥120 mL/kg/ day of expressed breast milk or formula by bottle (up to discharge to home).
