# Supplementary material for: Diazoxide for Severe or Recurrent Neonatal Hypoglycemia: A Randomized Clinical Trial
Source: JAMA Netw Open. 2024 Jun 13;7(6):e2415764. doi: 10.1001/jamanetworkopen.2024.15764 (PMC11177163; doi:10.1001/jamanetworkopen.2024.15764)
Supplement: Supplement 2. — eFigure 1. Intravenous Dextrose Delivery After Commencing Intervention eFigure 2. Resolution of Hypoglycemia, Post Hoc Definition eFigure 3. Insulin to Glucose Ratio at 36 Hours After Commencing Intervention eFigure 4. Interstitial Glucose After Last Intervention Dose eFigure 5. Interstitial Glucose Immediately After Intervention Loading Dose eFigure 6. Interstitial Glucose From Commencement of Intervention eTable 1. Estimated Difference in Interstitial Glucose Between Diazoxide vs Placebo Groups eFigure 7. Interstitial Glucose Among Neonates Born Small or Large for Gestational Age From Commencement of Intervention eTable 2. Estimated Difference in Interstitial Glucose Between Diazoxide vs Placebo Groups Among Neonates Born Small or Large for Gestational Age [file jamanetwopen-e2415764-s002.pdf]

Supplementary Online Content

Laing D, Walsh EPG, Alsweiler JM, et al. Diazoxide for severe or recurrent neonatal hypoglycemia: a randomized clinical trial. *JAMA Netw Open*. 2024;7(6):e2415764. doi:10.1001/jamanetworkopen.2024.15764

eFigure 1. Intravenous Dextrose Delivery After Commencing Intervention

eFigure 2. Resolution of Hypoglycemia, Post Hoc Definition

eFigure 3. Insulin to Glucose Ratio at 36 Hours After Commencing Intervention

eFigure 4. Interstitial Glucose After Last Intervention Dose

eFigure 5. Interstitial Glucose Immediately After Intervention Loading Dose

eFigure 6. Interstitial Glucose From Commencement of Intervention

eTable 1. Estimated Difference in Interstitial Glucose Between Diazoxide vs Placebo Groups

eFigure 7. Interstitial Glucose Among Neonates Born Small or Large for Gestational Age From Commencement of Intervention

eTable 2. Estimated Difference in Interstitial Glucose Between Diazoxide vs Placebo Groups, Among Neonates Born Small or Large for Gestational Age

This supplementary material has been provided by the authors to give readers additional information about their work.

© 2024 Laing D et al. *JAMA Network Open*.

eFigure 1: Intravenous dextrose delivery after commencing intervention

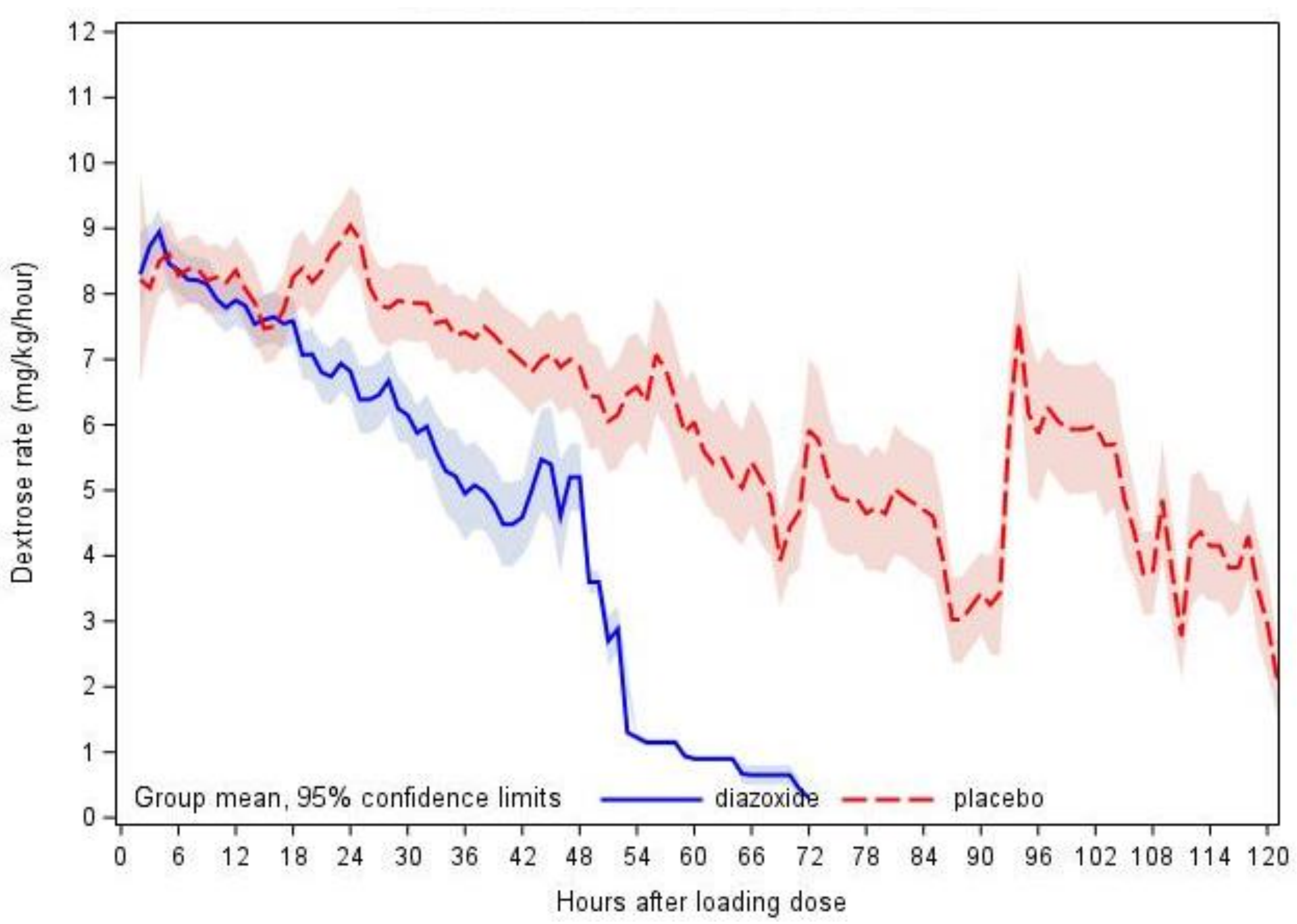

At the trial commencement, 25 infants in the diazoxide group and 23 in the placebo group were receiving intravenous fluids.

© 2024 Laing D et al. *JAMA Network Open*.

eFigure 2: Resolution of hypoglycemia, post hoc definition

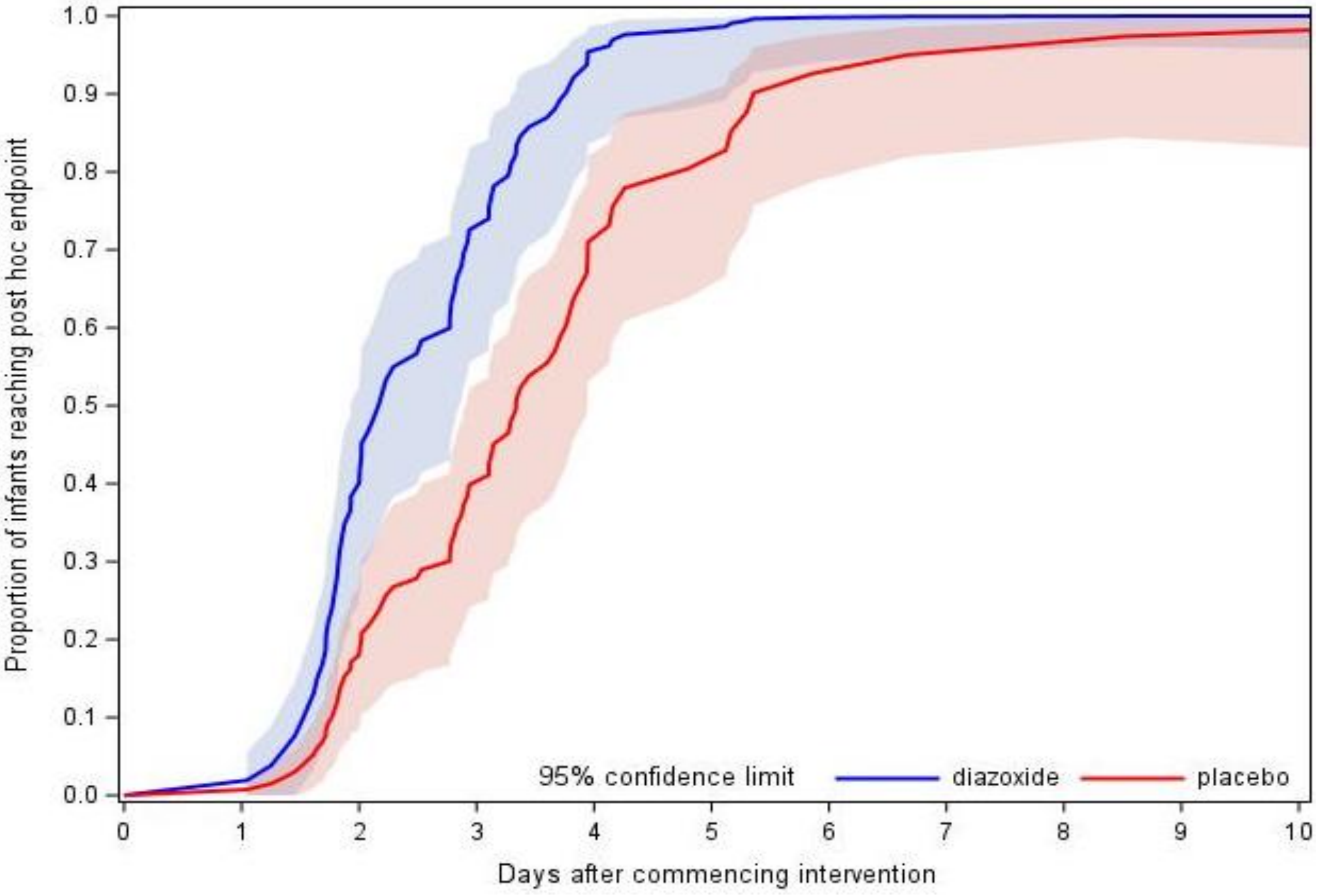

Resolution of hypoglycemia re-defined as enteral bolus feeding without intravenous fluids for ≥24 hours with no further hypoglycemia. Adjusted hazards ratio 2.60, 95% CI 1.53, 4.46. Hodges-Lehmann estimation of the median time (95% CI) 2.2 (1.9, 2.8) days with diazoxide intervention vs. 3.3 (2.8, 4.3) days with placebo.

© 2024 Laing D et al. *JAMA Network Open*.

eFigure 3: Insulin to glucose ratio at 36 hours after commencing intervention

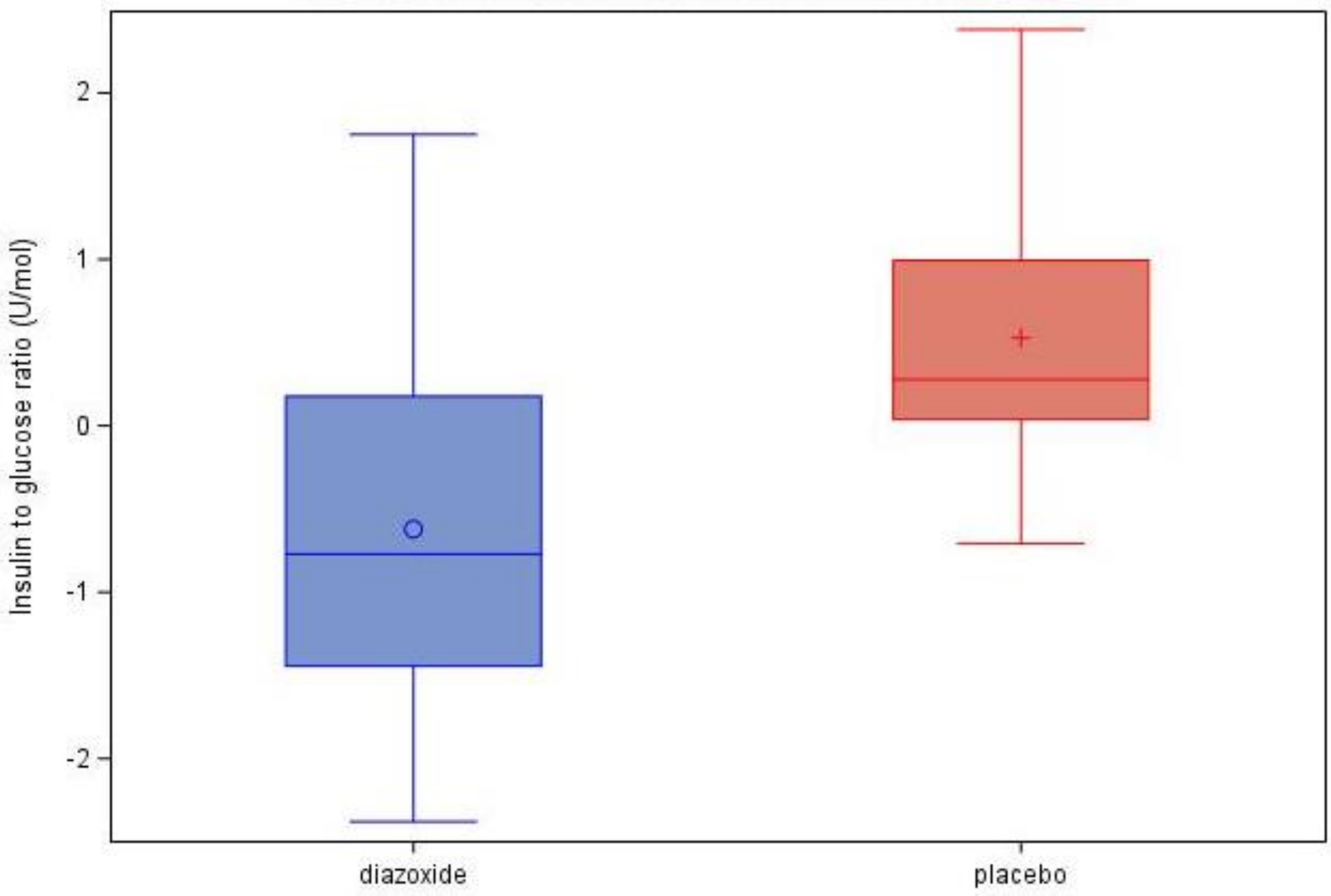

Only infants with a blood glucose measurement collected within 15 minutes of the insulin sample were included in this analysis (diazoxide N=14, placebo N=14). Adjusted ratio of geometric means 0.32, 95% CI 0.15, 0.65.

© 2024 Laing D et al. *JAMA Network Open*.

eFigure 4: Interstitial glucose after last intervention dose

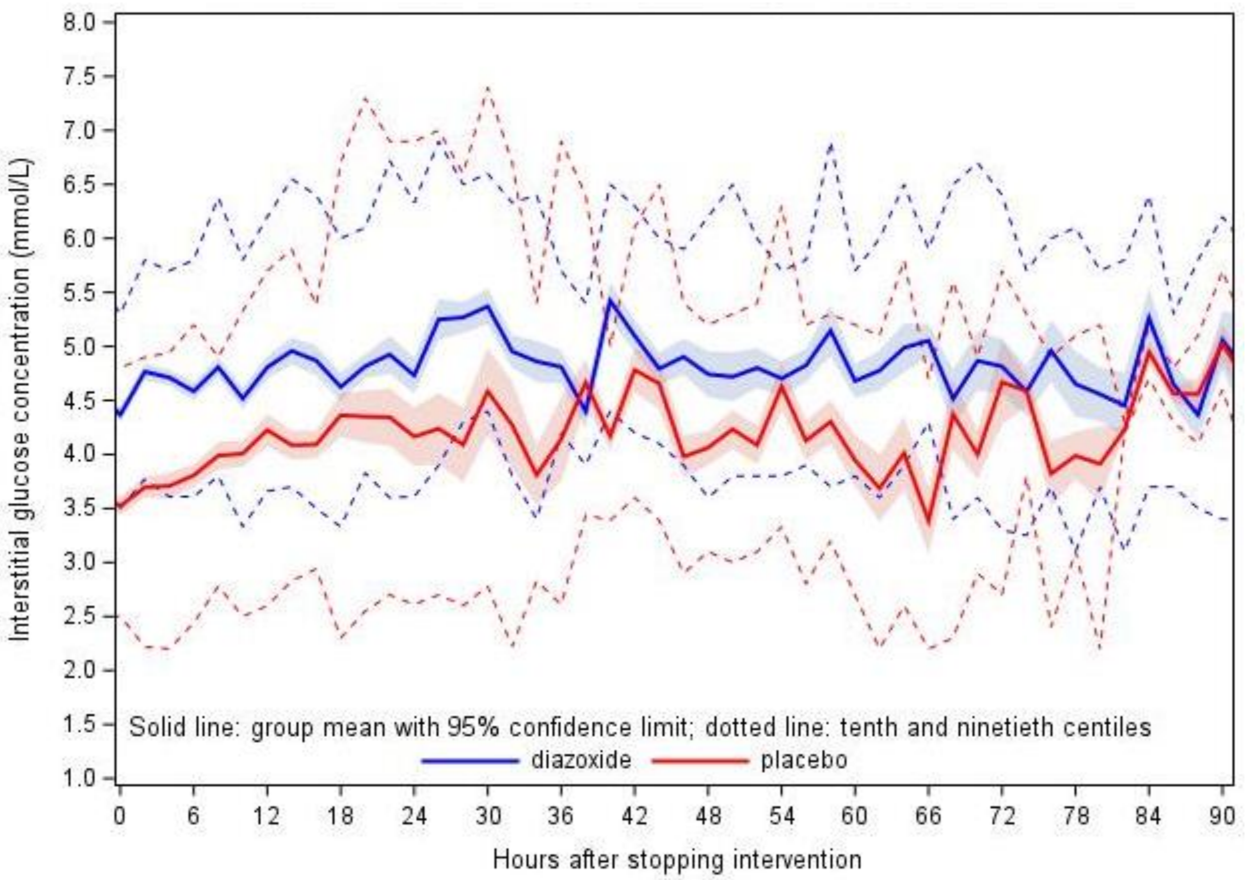

Interstitial glucose concentration measured using Medtronic Guardian Connect and Enlite-3 sensor (diazoxide N=26, placebo N=24). To convert mmol/L to mg/dL, divide by 0.0555.

© 2024 Laing D et al. *JAMA Network Open*.

eFigure 5: Interstitial glucose immediately after intervention loading dose

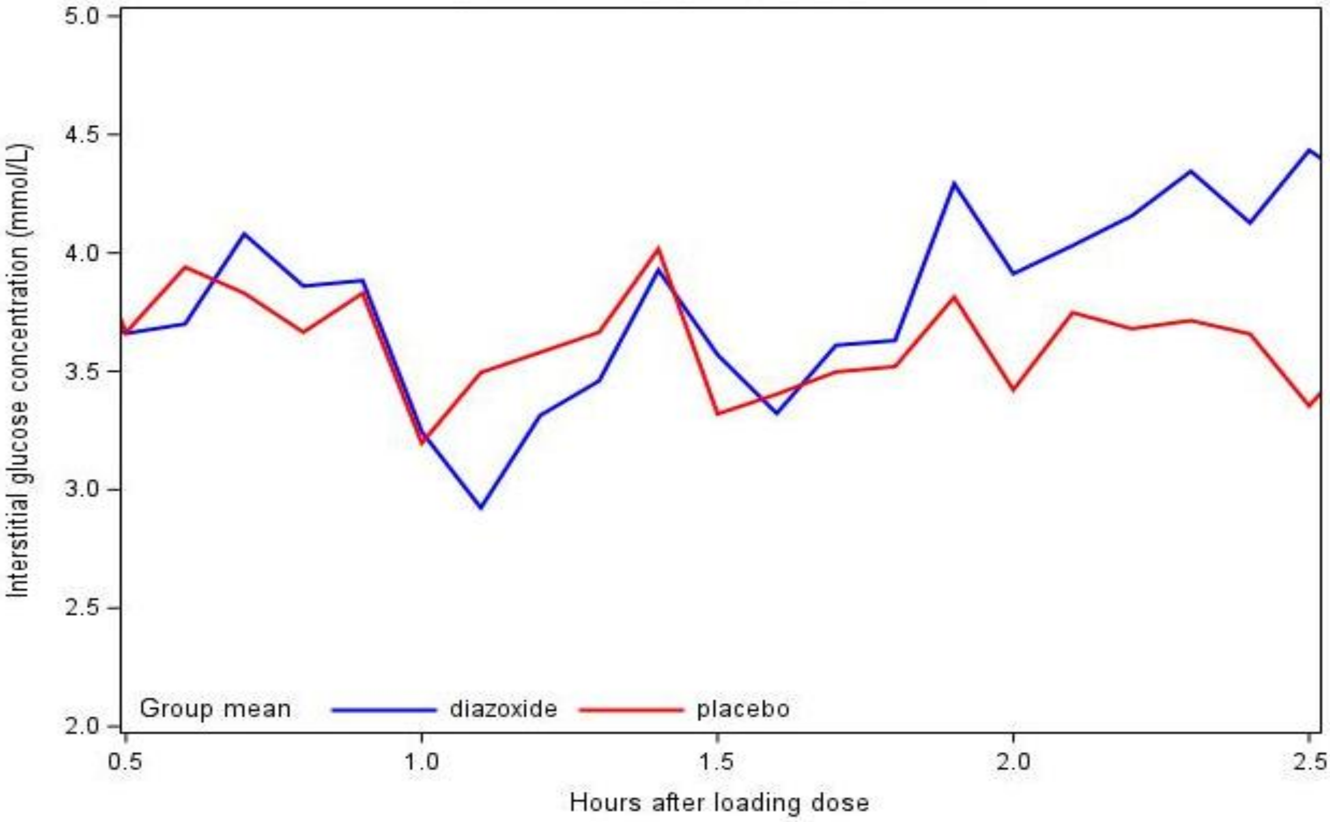

Interstitial glucose concentration measured using Medtronic Guardian Connect and Enlite-3 sensor (diazoxide N=26, placebo N=24). Where possible, the sensor was placed before the loading dose; however, because it has a two-hour wetting phase, fewer infants contributed data in the first 1 to 2 hours and thus only mean values are displayed. Separation of mean interstitial glucose starts to occur from around 1.5 hours and a 0.6 mmol/L difference between groups was evident from 2 to <6 hours (see Table S1). To convert mmol/L to mg/dL, divide by 0.0555.

© 2024 Laing D et al. *JAMA Network Open*.

eFigure 6: Interstitial glucose from commencement of intervention

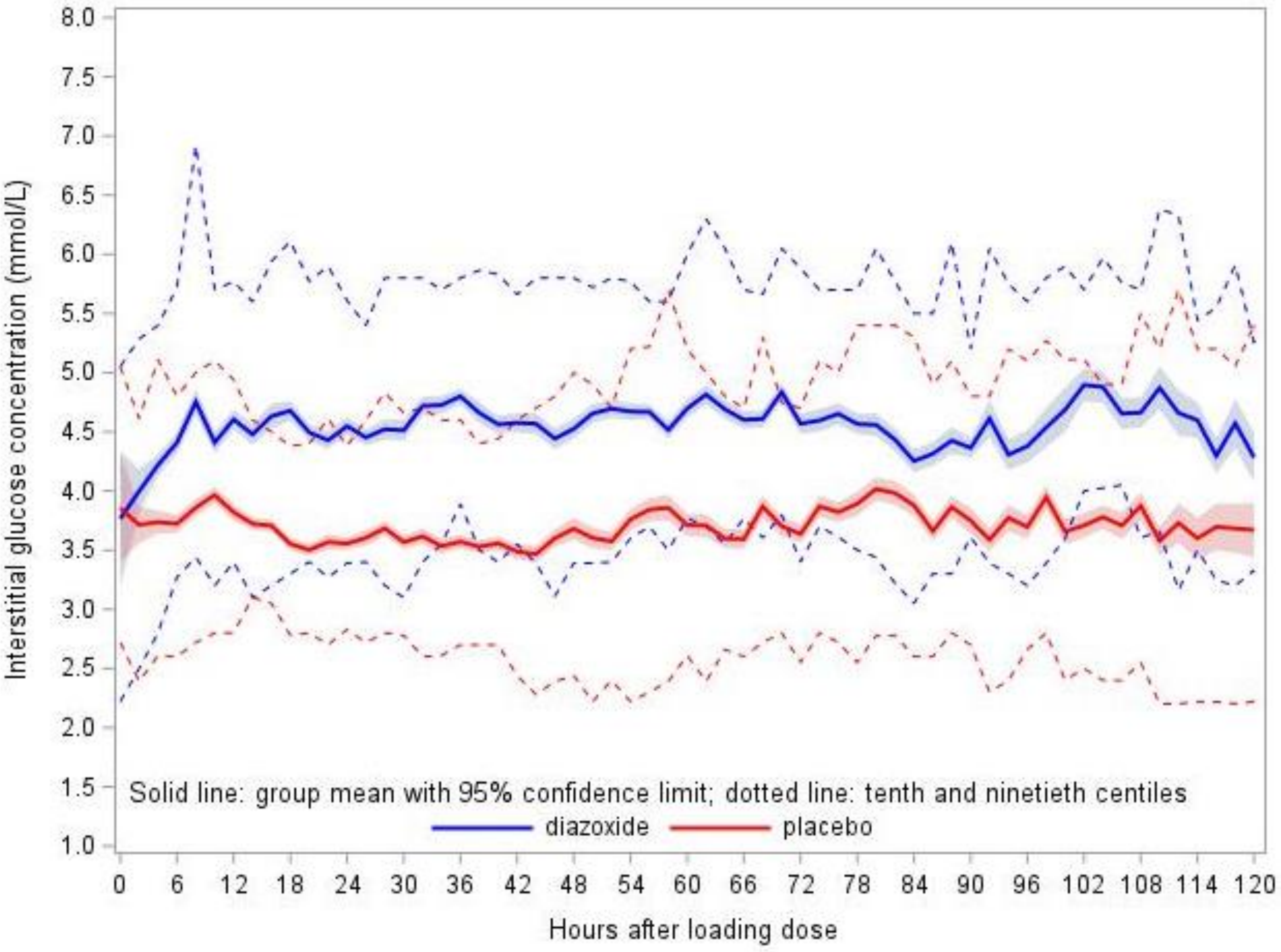

Interstitial glucose concentration measured using Medtronic Guardian Connect and Enlite-3 sensor (diazoxide N=26, placebo N=24). To convert mmol/L to mg/dL, divide by 0.0555.

© 2024 Laing D et al. *JAMA Network Open*.

eTable 1: Estimated difference in interstitial glucose between diazoxide vs. placebo groups

Time interval after loading dose (hours)	Mean difference (95% CI) mmol/L
<2	-0.4 (-1.2, 0.5)
2 to <6	0.6 (0.1, 1.1)
6 to <12	0.7 (0.2, 1.2)
12 to <24	0.9 (0.5, 1.3)
>24 to <120	0.8 (0.5, 1.0)

Repeated measures analysis: diazoxide N=26; placebo N=24. To convert mmol/L to mg/dL, divide by 0.0555.

© 2024 Laing D et al. *JAMA Network Open*.

eFigure 7: Interstitial glucose among neonates born small or large for gestational age from commencement of intervention

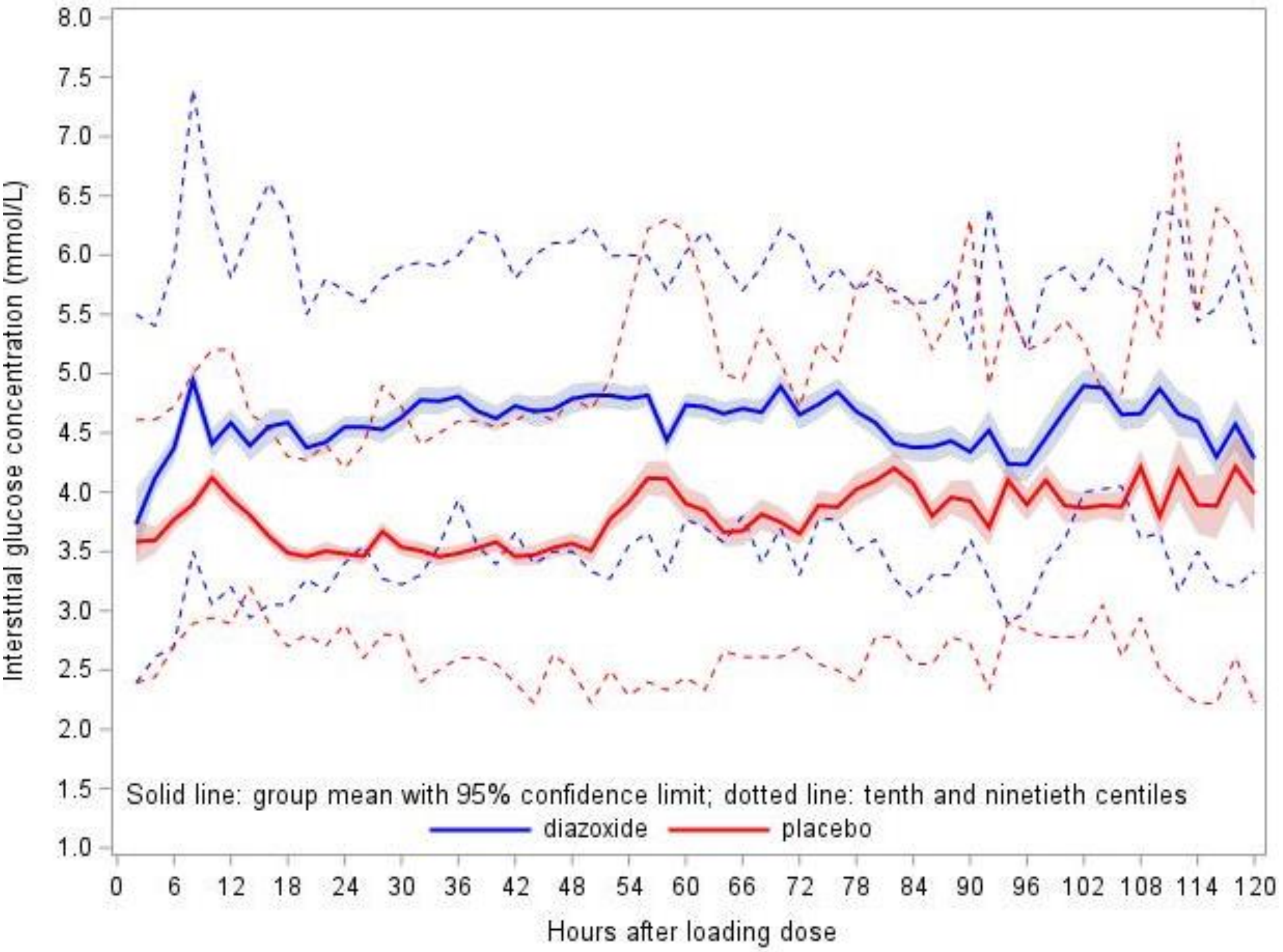

Interstitial glucose concentration measured using Medtronic Guardian Connect and Enlite-3 sensor (diazoxide N=17, placebo N=15). The analysis includes only infants with a customised birthweight <10th or >90th centile. To convert mmol/L to mg/dL, divide by 0.0555.

© 2024 Laing D et al. *JAMA Network Open*.

eTable 2: Estimated difference in interstitial glucose between diazoxide vs. placebo groups, among neonates born small or large for gestational age

Time interval after loading dose (hours)	Mean difference (95% CI) mmol/L
<2	-0.1 (-1.4, 1.1)
2 to <6	0.4 (-0.2, 1.1)
6 to <12	0.6 (-0.1, 1.4)
12 to <24	0.9 (0.3, 1.4)
>24 to <120	0.6 (0.3, 1.0)

Repeated measures analysis: diazoxide N=17; placebo N=15. To convert mmol/L to mg/dL, divide by 0.0555.

© 2024 Laing D et al. *JAMA Network Open*.
