# Supplementary material for: Diazoxide for Severe or Recurrent Neonatal Hypoglycemia: A Randomized Clinical Trial
Source: JAMA Netw Open. 2024 Jun 13;7(6):e2415764. doi: 10.1001/jamanetworkopen.2024.15764 (PMC11177163; doi:10.1001/jamanetworkopen.2024.15764)
Supplement: Supplement 3. — Data Sharing Statement [file jamanetwopen-e2415764-s003.pdf]

Data Sharing Statement

Laing. Diazoxide for Severe or Recurrent Neonatal Hypoglycemia. *JAMA Netw Open*. Published June 13, 2024. doi:10.1001/jamanetworkopen.2024.15764

Data
Data available: Yes
Data types: Other (please specify)
Additional Information: Anonymised data may be shared with external researchers upon request, by contacting the corresponding author.
How to access data: Anonymised data may be shared with external researchers upon request, by contacting the corresponding author, Christopher JD McKinlay, c.mckinlay@auckland.ac.nz
When available: With publication

Supporting Documents
Document types: None

Additional Information
Who can access the data: Anonymised data may be shared with external researchers upon request, by contacting the corresponding author.
Types of analyses: For purposes related to the study aims.
Mechanisms of data availability: After approval of a proposal with a signed data access agreement.
Any additional restrictions: Local ethical/institutional approval. Provision of a suitable study protocol.
